# Association of multimorbidity with mortality after stroke stratified by age, severity, etiology, and prior disability

**DOI:** 10.1177/17474930231210397

**Published:** 2023-11-22

**Authors:** Matthew B Downer, Ramon Luengo-Fernandez, Lucy E Binney, Sergei Gutnikov, Louise E Silver, Aubretia McColl, Peter M Rothwell

**Affiliations:** Wolfson Centre for the Prevention of Stroke and Dementia, Nuffield Department of Clinical Neurosciences, Wolfson Building—John Radcliffe Hospital, University of Oxford, Oxford, UK

**Keywords:** Chronic disease, epidemiology, long-term outcomes, multimorbidity, prognosis, post-stroke mortality

## Abstract

**Background:**

Multimorbidity is common in patients with stroke and is associated with increased medium- to long-term mortality, but its value for clinical decision-making and case-mix adjustment will depend on other factors, such as age, stroke severity, etiological subtype, prior disability, and vascular risk factors.

**Aims:**

In the absence of previous studies, we related multimorbidity to long-term post-stroke mortality with stratification by these factors.

**Methods:**

In patients ascertained in a population-based stroke incidence study (Oxford Vascular Study; 2002–2017), we related pre-stroke multimorbidity (weighted/unweighted Charlson comorbidity index (CCI)) to all-cause/vascular/non-vascular mortality (1/5/10 years) using regression models adjusted/stratified by age, sex, predicted early outcome (THRIVE score), stroke severity (NIH stroke scale (NIHSS)), etiology (Trial of Org 10172 in Acute Stroke Treatment (TOAST)), premorbid disability (modified Rankin Scale (mRS)), and non-CCI risk factors (hypertension, hyperlipidemia, atrial fibrillation, smoking, deprivation, anxiety/depression).

**Results:**

Among 2454 stroke patients (M/SD age: 74.1/13.9 years; 48.9% male; M/SD NIHSS: 5.7/7.0), 1375/56.0% had ⩾ 1 CCI comorbidity and 685/27.9% had ⩾ 2. After age/sex adjustment, multimorbidity (unweighted CCI ⩾ 2 vs 0) predicted (all ps < 0.001) mortality at 1 year (aHR = 1.57, 95% CI = 1.38–1.78), 5 years (aHR = 1.73, 95% CI = 1.53–1.96), and 10 years (aHR = 1.79, 95% CI = 1.58–2.03). Although multimorbidity was independently associated with premorbid disability (mRS > 2: aOR = 2.76, 2.13–3.60) and non-CCI risk factors (hypertension: 1.56, 1.25–1.95; hyperlipidemia: 2.58, 2.03–3.28; atrial fibrillation: 2.31; 1.78–2.98; smoking: 1.37, 1.01–1.86), it predicted death after adjustment for all measured confounders (10-year-aHR = 1.56, 1.37–1.78, p < 0.001), driven mainly by non-vascular death (aHR = 1.89, 1.55–2.29). Predictive value for 10-year all-cause death was greatest in patients with lower expected early mortality: lower THRIVE score (p_int_ < 0.001), age < 75 years (aHR = 2.27, 1.71–3.00), NIHSS < 5 (1.84, 1.53–2.21), and lacunar stroke (3.56, 2.14–5.91). Results were similar using the weighted CCI.

**Conclusion:**

Pre-stroke multimorbidity is highly prevalent and is an independent predictor of death after stroke, supporting its inclusion in case-mix adjustment models and in informing decision-making by patients, families, and carers. Prediction in younger patients and after minor stroke, particularly for non-vascular death, suggests potential clinical utility in targeting interventions that require survival for 5–10 years to achieve a favorable risk/benefit ratio.

**Data access statement:**

Data requests will be considered by the Oxford Vascular Study (OXVASC) Study Director (P.M.R.-peter.rothwell@ndcn.ox.ac.uk).

## Introduction

Multimorbidity, defined as the co-occurrence of two or more long-term conditions,^1^ is common in patients who have a stroke.^2^ In the general population, multimorbidity is associated with increased short- and long-term mortality and lower health-related quality of life.^3–7^ With rapidly aging populations and increasing obesity, the prevalence of multimorbidity is predicted to continue to rise in the coming decades.^3,8–10^

Studies have shown that multimorbidity predicts increased mortality after stroke, with most using the Charlson comorbidity index (CCI), which can be routinely coded using administrative data, to predict all-cause death.^2,11–14^ However, the added value of multimorbidity for case-mix adjustment and clinical prognostication will likely depend on the mortality rate expected on the basis of other factors, such as age, stroke severity, etiological subtype, prior disability, and vascular risk factors, and might differ in relation to the cause of death. Although multimorbidity might be expected to be associated with older age and more severe strokes, predictive value might be limited in this setting by ceiling effects related to the high underlying short- and medium-term mortality.

In the absence of previous studies with stratification by stroke severity or related factors, we aimed to determine the predictive value of multimorbidity for post-stroke mortality (all-cause, vascular, and non-vascular) with stratification by predicted early outcome, age, premorbid disability, stroke severity, etiological subtype, and vascular risk factors. We also aimed to determine the extent to which these and other factors that may be associated with multimorbidity, such as deprivation, depression/anxiety, and other risk factors not included in the CCI (hypertension, hyperlipidemia, atrial fibrillation, and smoking) might confound the association with post-stroke mortality.

## Methods

### Study population and background

The Oxford Vascular Study (OXVASC) is a population-based study of all acute vascular events in a population of 94,973 persons covered by nine general practices across Oxfordshire, United Kingdom.^15–18^ The current sample included all first-in-study strokes ascertained between April 1, 2002 and March 31, 2017. Briefly, ascertainment included a daily rapid access transient ischemic attack (TIA)/stroke clinic for patients with suspected TIA or stroke who were registered with a partnering general practice, daily searches of hospital admissions to emergency departments, relevant wards, and death records, and monthly searches of death certificates, coroner reports, GP diagnostic codes, and brain/vascular imaging referrals. Additional details on ascertainment methodology and underlying study population are published elsewhere.^16–18^ OXVASC has received approval from the local health research ethics committee (OREC A: 05/Q1604/70).

Patients were assessed by study physicians within 1–2 days after the stroke. We obtained informed consent from patients or assent from relatives. Initial assessment included a full, detailed clinical history and examination, including event severity at the time of assessment (NIH Stroke Scale score (NIHSS)),^19^ risk factors, comorbidities, place of residence, and premorbid disability (modified Rankin Scale (mRS)).^20^ Data on premorbid comorbidities were supplemented by review of primary and secondary care (including paper records and administrative coding data) and quantified using the CCI, which is the most commonly used multimorbidity index in stroke research^2^ and it has been previously validated for use in stroke outcome studies.^13^ Place of residence, including post code, was used to quantify the level of deprivation through English Indices of Multiple Deprivation (IMD), and categorized by quartile (1 = *most affluent*, 4 = *most deprived*).^21^

All cases were reviewed by the senior study neurologist (P.M.R.) and etiological subtype classified using the Trial of Org 10172 in Acute Stroke Treatment (TOAST) system.^22^ First-in-study stroke patients were followed-up face-to-face by the OXVASC research team at 1, 3, 6 and 12 months and 5 and 10 years. Follow-up was supplemented by review of both GP and hospital records, via access to centralized UK death records, and by continued day-to-day ascertainment of recurrent vascular events and deaths. Deaths were classified as either vascular or non-vascular using International Classification of Disease-10th Revision (ICD-10) codes.

Data requests will be considered by the senior investigator (peter.rothwell@ndcn.ox.ac.uk).

### Statistical analyses

Follow-up was completed to December 2020 or death if earlier, but analyses were truncated at 10 years due to right-hand censoring or prior death. Patients with incomplete medical history at baseline were excluded from analyses and no data were imputed.

Patients were classified as living with either no comorbidity (CCI = 0), one comorbidity (CCI = 1), or multimorbidity (CCI ⩾ 2), using both the weighted and unweighted versions of the index. The unweighted CCI is a count of the number of comorbidities, while the weighted CCI assigns weights (i.e. points) to each comorbidity.^13^ The individual components and respective weights of comorbidities in the CCI are outlined in the Supplemental Appendix (Table S1). The principal set of analyses compared CCI ⩾ 2 (multimorbidity) versus CCI = 0 (no comorbidity), using both the unweighted and weighted versions of the CCI.

Continuous data were reported as mean (standard deviation; SD) or median (interquartile range; IQR), as appropriate, and categorical data as counts (%). Differences between multimorbidity groups were explored using one-way analysis of variance (ANOVA) for continuous variables, chi-squared for categorical variables, and Kruskal–Wallis tests to assess differences in median values, as appropriate.

To determine the associates of multimorbidity, crude and adjusted logistic regression models were used to obtain odds ratios (ORs) with 95% confidence intervals (95% CIs) for age (⩾ 85 and 75–84 vs < 75 years), premorbid disability (mRS ⩾ 3 vs 0–2), smoking (current or ex vs never), deprivation (IMD score highest quartile vs lowest), stroke severity (NIHSS ⩾ 10 and 5–9 vs 0–4), and other measured risk factors (history of depression or anxiety, hypertension, hyperlipidemia, and atrial fibrillation). All analyses were run unadjusted (Model 1), with age and sex (Model 2), and with all other variables above (Model 3).

Cox proportional hazard models were used to obtain crude/adjusted hazard ratios for the association between multimorbidity and risk of death at 1-, 5-, and 10 years post-stroke, with the same hierarchy of adjustment listed above in addition to a further model, including age, sex, and one of the variables of interest. Analyses were done for all-cause, vascular, and non-vascular death, and with stratification by age, stroke severity, premorbid disability, smoking history, deprivation, vascular risk factors, and TOAST subtype.

Additional analyses were stratified by age (⩾/< 75 years) and sex. To stratify analyses of the predictive value of multimorbidity by likely early outcome, we classified all patients using the THRIVE score.^23^ The score, which includes age, NIHSS, and three vascular risk factors (diabetes, hypertension, and atrial fibrillation), was developed to predict 90-day mortality and functional outcome after stroke,^23^ and has been externally validated.^24^ Patients are categorized into three risk groups (0–2: low, 3–5: moderate, and 6–9: high). As THRIVE was based on NIHSS on presentation and we ascertained NIHSS in the sub-acute phase (typically 24-h post-event), we used both the original THRIVE NIHSS scoring (NIHSS ⩽ 10: 0 points, 11–20: 2 points, ⩾ 21: 4 points) and an adjusted version to take into account early recovery (NIHSS ⩽ 4: 0 points, 5–9: 2 points, ⩾ 10: 4 points). We then stratified patients by THRIVE group and obtained crude/adjusted hazard ratios for multimorbidity (CCI ⩾ 2 vs 0; weighted/unweighted) for all-cause mortality at 1- and 10 years post-stroke. Kaplan–Meier curves for all-cause mortality were plotted by multimorbidity group with log-rank tests, stratified by THRIVE group. We tested the hypothesis that predictive value of multimorbidity would fall as the THRIVE score increased by including an interaction term in a Cox model relating CCI to all-cause mortality.

Possible violations of the proportional-hazards assumption were assessed by inspection of log–log plots and chi-square tests of Schoenfeld residuals. Statistical analyses were performed using Stata (V16; College Station, USA), and significance level was set at p-value less than 0.05 for all analyses.

## Results

Among 2540 patients with a first-in-study stroke ascertained from 2002 to 2017, 86 (3.4%) were excluded due to incomplete baseline data. Therefore, 2454 were included in the final cohort (M/SD age = 74.4/13.9 years; 1201/48.9% male; median NIHSS = 3, IQR 1–8). Of these, 1375 patients (56.0%) had at least one prior CCI comorbidity and 685 (27.9%) had multimorbidity (⩾ 2 CCI comorbidities; Table 1), with history of solid cancer (n = 339, 13.8%), diabetes without end organ damage (n = 280, 11.4%), and previous myocardial infarction (n = 280, 11.4%) being most frequent (Supplemental Appendix-Tables S1 and S2).

**Table 1.** Baseline clinical/demographic variables of 2454 first-in-study stroke patients, stratified by CCI group (unweighted).

Clinical/demographic variables	CCI (unweighted)			Full cohort (n = 2454)
	0 (n = 1079)	1 (n = 690)	⩾ 2 (n = 685)	
Age^***^	71.00 (15.65)	75.70 (12.77)	78.50 (10.39)	74.41 (13.92)
Male sex	537 (49.8%)	316 (45.8%)	348 (50.8%)	1201 (48.9%)
Premorbid mRS^***^	1 (0–1)	1 (0-3)	2 (1–3)	1 (0–2)
Index of multiple deprivation^**^	9.30 (5.89–13.06)	9.49 (5.78–13.75)	10.00 (5.97–14.02)	9.67 (5.78–13.75)
Smoking history^***^				
Non-smoker	195 (18.1%)	95 (13.8%)	90 (13.1%)	380 (15.5%)
Ex-smoker	374 (34.7%)	283 (41.0%)	314 (45.8%)	971 (39.6%)
Current smoker	510 (47.3%)	312 (45.2%)	281 (41.0%)	1103 (44.9%)
History of depression or anxiety	240 (22.2%)	158 (22.9%)	168 (24.5%)	566 (23.1%)
Hypertension^***^	555 (51.4%)	436 (63.2%)	485 (70.8%)	1476 (60.1%)
Hyperlipidemia^***^	201 (18.6%)	188 (27.2%)	253 (36.9%)	642 (26.2%)
Atrial fibrillation^***^	156 (14.5%)	128 (18.6%)	237 (34.6%)	521 (21.2%)
Sub-acute NIH stroke scale^***^	2 (1–7)	3 (1–9)	3 (1–9)	3 (1–8)
Etiological subtype^***^				
Intracerebral hemorrhage	107 (9.9%)	67 (9.7%)	47 (6.9%)	221 (9.0%)
Subarachnoid hemorrhage	56 (5.2%)	30 (4.3%)	9 (1.3%)	95 (3.9%)
Cardioembolic	227 (21.0%)	179 (25.9%)	238 (34.7%)	644 (26.2%)
Large artery disease	92 (8.5%)	60 (8.7%)	63 (9.2%)	215 (8.8%)
Small vessel disease	150 (13.9%)	81 (11.7%)	45 (6.6%)	276 (11.2%)
Undetermined	280 (25.9%)	128 (18.6%)	114 (16.6%)	522 (21.3%)
Unknown	108 (10.0%)	105 (15.2%)	119 (17.4%)	332 (13.5%)
Multiple	31 (2.9%)	27 (3.9%)	37 (5.4%)	95 (3.9%)
Other	28 (2.6%)	13 (1.9%)	13 (1.9%)	54 (2.2%)

CCI: Charlson comorbidity index; NIH: National Institutes of Health; mRS: modified Rankin scale.

* p < 0.05, ^**^p < 0.01, ^***^p < 0.001 between CCI groups.

Data expressed as count (%), M (SD), or median (IQR).

An analogous table stratified by weighted CCI groups is found in the Supplemental Appendix.

Pre-stroke multimorbidity was most strongly associated with premorbid disability (unweighted CCI ⩾ 2 vs 0: OR = 3.37, 95% CI = 2.67–4.21 for mRS ⩾ 3; weighted CCI: 2.76, 2.22–3.45; both p < 0.001), with little diminution after adjustment for all other variables (unweighted: aOR = 2.76, 2.13–3.60; weighted: aOR = 2.15, 1.68–2.76, both p < 0.001), whereas NIHSS was not independently associated (Table 2). Hypertension, hyperlipidemia, and atrial fibrillation were each independently associated with multimorbidity, using both the unweighted and weighted CCI, whereas deprivation and smoking were only associated with the unweighted index, and history of depression/anxiety disorders was not associated (Table 2; Supplemental Appendix-Table S3 and Figures S4 to S9). Associations tended to be stronger in younger patients (Supplemental Appendix-Table S8).

**Table 2.** Univariate and adjusted associations between multimorbidity and various premorbid/baseline variables.

Premorbid or baseline characteristic	OR (95% CI) for CCI ⩾ 2 versus 0 (unweighted/counts)		
	Model 1	Model 2	Model 3
	Crude	+ Age/sex	+ All
Age^a^ (years)			
<75	1.00	1.00	1.00
75–84	2.47 (1.96–3.10)^***^	2.54 (2.02–3.19)^***^	1.75 (1.35–2.27)^***^
⩾85	2.63 (2.05–3.38)^***^	2.79 (2.16–3.60)^***^	1.55 (1.14–2.10)^**^
Sub-acute phase NIHSS^b^			
0–4	1.00	1.00	1.00
5–9	1.35 (1.03–1.77)^*^	1.12 (0.85–1.48)	1.03 (0.77–1.39)
⩾10	1.43 (1.13–1.82)^**^	1.19 (0.93–1.52)	0.95 (0.73–1.25)
Premorbid disability (mRS)^b^			
0–2	1.00	1.00	1.00
⩾3	3.37 (2.67–4.21)^***^	2.75 (2.14–3.52)^***^	2.76 (2.13–3.60)^***^
Deprivation level^a^			
Least deprived	1.00	1.00	1.00
Most deprived	1.32 (1.01–1.73)^*^	1.48 (1.11–1.96)^*^	1.37 (1.01–1.86)^*^
Smoking status^a^			
Never smoked	1.00	1.00	1.00
Ever smoked	1.28 (1.06–1.56)^*^	1.39 (1.13–1.72)^**^	1.35 (1.08–1.70)^**^
Depression or anxiety^b^	1.14 (0.91–1.42)	1.29 (1.02–1.64)^*^	1.11 (0.87–1.44)
Hypertension^b^	2.29 (1.87–2.81)^***^	1.90 (1.54–2.34)^***^	1.56 (1.25–1.95)^***^
Hyperlipidemia^b^	2.56 (2.06–3.18)^***^	2.75 (2.19–3.44)^***^	2.58 (2.03–3.28)^***^
Atrial fibrillation^b^	3.13 (2.48–3.95)^***^	2.46 (1.93–3.12)^***^	2.31 (1.78–2.98)^***^

CI: confidence interval; CCI: Charlson comorbidity index; NIHSS: National Institutes of Health Stroke Scale; IMD: index of multiple deprivation; mRS: modified Rankin scale.

a Predictor variables.

b Outcome variables.

Expressed as OR (95% CI) for CCI ⩾ 2 versus 0.

Model 3 included age, sex, and all other listed variables to the exception of the variable of interest.

* p < 0.05; ^**^p < 0.01; ^***^p < 0.001 between CCI groups.

An analogous table using the weighted CCI is provided in the Supplemental Appendix.

After adjustment for age and sex, multimorbidity (unweighted CCI ⩾ 2 vs 0) predicted (all ps < 0.001) post-stroke mortality at 1 year (unweighted: aHR = 1.57, 95% CI = 1.38–1.78; weighted: 1.48, 1.31–1.67), 5 years (unweighted: aHR = 1.73, 1.53–1.96; weighted: 1.60, 1.42–1.81), and 10 years (unweighted: aHR = 1.79, 1.58–2.03; weighted: 1.67, 1.48–1.88). After further adjustment for all measured confounders, multimorbidity was still associated (p < 0.001) with all-cause mortality at 1 year (unweighted: aHR = 1.36, 1.19–1.56; weighted: 1.33, 1.17–1.50), 5 years (unweighted: aHR = 1.49, 1.31–1.71; weighted: 1.44, 1.28–1.63), and 10 years follow-up (unweighted: aHR = 1.56, 1.37–1.78; weighted: 1.51, 1.33–1.71; Table 3). However, after age and sex, adjustment for premorbid mRS had the greatest attenuating effect on these associations (Table 3; Supplemental Appendix-Tables S4, S5, S7 and S10). Again, associations were strongest in younger patients (Supplemental Appendix-Table S9).

**Table 3.** Univariate and adjusted associations between multimorbidity and all-cause, vascular, and non-vascular mortality up to 10 years post-stroke.

Model No.	Adjustments	Hazard ratios (95% CI) for CCI ⩾ 2 versus 0 (unweighted/counts)				
		1 year All-cause	5 years All-cause	10 years All-cause	10 years Vascular	10 years Non-vascular
Model 1	Crude	2.04 (1.80–2.31)	2.30 (2.03–2.61)	2.41 (2.12–2.73)	2.02 (1.67–2.43)	2.72 (2.26–3.28)
Model 2	Age, sex	1.57 (1.38–1.78)	1.73 (1.53–1.96)	1.79 (1.58–2.03)	1.55 (1.28–1.87)	2.00 (1.66–2.41)
Model 3A	+ IMD	1.55 (1.37–1.76)	1.71 (1.51–1.94)	1.78 (1.57–2.01)	1.53 (1.27–1.85)	2.00 (1.66–2.41)
Model 3B	+ Smoking	1.57 (1.38–1.78)	1.73 (1.53–1.96)	1.79 (1.58–2.03)	1.55 (1.28–1.87)	2.00 (1.66–2.41)
Model 3C	+ Depression/anxiety	1.58 (1.39–1.79)	1.74 (1.53–1.97)	1.80 (1.58–2.04)	1.57 (1.30–1.89)	2.01 (1.66–2.42)
Model 3D	+ Prior hypertension	1.57 (1.38–1.78)	1.73 (1.52–1.96)	1.80 (1.59–2.04)	1.52 (1.26–1.84)	2.08 (1.72–2.51)
Model 3E	+ Prior Hyperlipidemia	1.56 (1.38–1.77)	1.74 (1.53–1.98)	1.82 (1.60–2.07)	1.52 (1.26–1.85)	2.10 (1.74–2.54)
Model 3F	+ Prior atrial fibrillation	1.50 (1.32–1.70)	1.66 (1.46–1.88)	1.72 (1.51–1.95)	1.45 (1.20–1.76)	1.95 (1.62–2.35)
Model 3G	+ NIHSS	1.55 (1.37–1.76)	1.73 (1.53–1.97)	1.79 (1.58–2.03)	1.50 (1.24–1.81)	2.02 (1.67–2.43)
Model 3H	+ mRS	1.42 (1.25–1.61)	1.54 (1.35–1.74)	1.59 (1.40–1.81)	1.37 (1.13–1.66)^a^	1.80 (1.49–2.17)
Model 4	+ All	1.36 (1.19–1.56)	1.49 (1.31–1.71)	1.56 (1.37–1.78)	1.25 (1.02–1.52)^b^	1.89 (1.55–2.29)

CI: confidence interval; CCI: Charlson comorbidity index; NIHSS: National Institutes of Health Stroke Scale; IMD: index of multiple deprivation; mRS: modified Rankin scale.

Expressed as hazard ratio (95% CI) for CCI ⩾ 2 versus 0.

All ps < 0.001, except ^a^(p = 0.001); ^b^(p = 0.032).

An analogous table using the weighted CCI is provided in the Supplemental Appendix.

Multimorbidity was most strongly predictive of non-vascular mortality (CCI ⩾ 2 vs 0: unweighted 10-year aHR = 1.89, 1.55–2.29; weighted: 1.84, 1.53–2.21, both p < 0.001; vascular mortality—unweighted-aHR = 1.25, 1.02–1.52, p = 0.032; weighted: 1.18, 0.98–1.42, p = 0.09; Table 3, Supplemental Appendix-Table S6 and Figure S1). The CCI components driving most of the association with non-vascular death were cancer, chronic obstructive pulmonary disease, chronic kidney disease, and peptic ulcer disease (Supplemental Appendix-Table S11).

**Figure 1.**
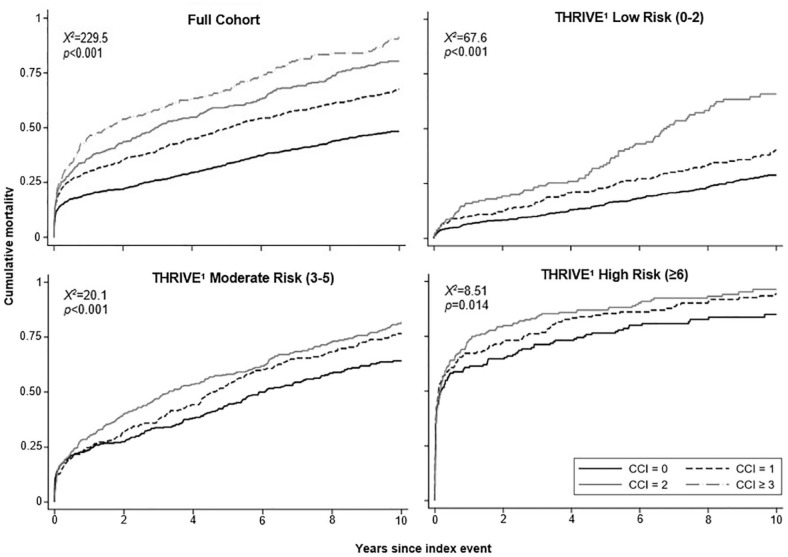
Kaplan–Meier curves for all-cause mortality up to 10 years post-stroke across multimorbidity groups (unweighted Charlson Index), in the overall cohort and stratified by adjusted THRIVE score. Log-rank tests shown in respective Kaplan–Meier plots. Original THRIVE scoring includes the following NIHSS categories (NIHSS ⩽ 10: 0 points, 11–20: 2 points, ⩾ 21: 4 points) based on NIHSS at presentation. This figure used adjusted THRIVE categories with altered NIHSS scoring (NIHSS ⩽ 4: 0 points, 5–9: 2 points, ⩾ 10: 4 points) as the present study included sub-acute phase NIHSS as opposed to NIHSS on presentation. Only three CCI (unweighted) categories are presented for the panels using THRIVE scores, with gray representing all patients with ⩾ 2 CCI comorbidities. Curves stratified by original THRIVE scores are found in the Supplemental Appendix.

The predictive value of CCI for 1-, 5- and 10-year all-cause death was greatest in patients predicted to have a good early outcome (p_int_ < 0.001 across THRIVE groups; Figure 1 and Supplemental Appendix-Table S12/Figures S2 and S3), with little difference between the weighted and unweighted CCI. For example, in the low-risk group using the original THRIVE scoring, multimorbidity predicted all-cause death at 1 year (unweighted: aHR = 1.68, 1.15–2.47; weighted: 1.65, 1.18–2.31, p < 0.01) and 10 years (unweighted: aHR = 2.11, 1.67–2.66; weighted: 1.71, 1.39–2.10, both p < 0.001). Results were similar using the THRIVE score with the adjusted NIHSS scoring (Supplemental Appendix-Table S12).

**Figure 2.**
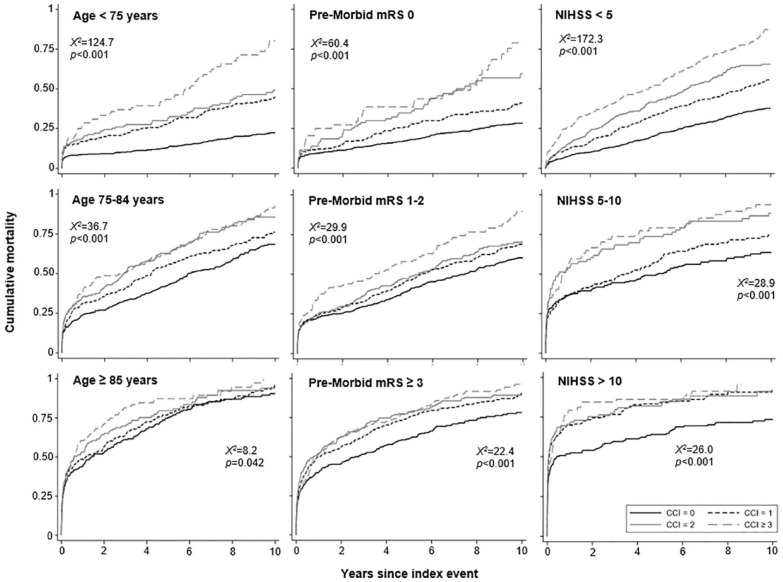
Kaplan–Meier curves for all-cause mortality up to 10 years post-stroke across multimorbidity groups, stratified by age, premorbid disability level, or stroke severity. Log-rank tests shown in respective Kaplan–Meier plots. Unweighted CCI groups were used.

Stratified analyses showed that CCI predicted all-cause death most strongly in younger patients (age < 75 years, crude HR for 10-year mortality = 3.46, 2.67–4.48), those with minor stroke (NIHSS < 5: 2.83, 2.38–3.37), and those without premorbid disability (mRS < 3: 2.26, 1.94–2.64, all ps < 0.001; Figure 2), but predictive value was largely unrelated to deprivation level, smoking history, depression/anxiety, hypertension, hyperlipidemia, and atrial fibrillation (Table 4). An apparently greater predictive power in men than women was largely explained by differences in M/SD age (males: 71.9/13.5; females: 76.9/13.9; Supplemental Appendix-Table S13).

**Table 4.** Univariate and adjusted hazard ratios for all-cause death at 10 years post-stroke, stratified by various premorbid/baseline variables.

Premorbid or baseline characteristic	Hazard ratio (95% CI) for CCI ⩾ 2 versus 0 (unweighted/counts)		
	Model 1	Model 2	Model 3
	Crude	+ Age/sex	+ All
Age (years)			
<75	3.46 (2.67–4.48)^***^	2.80 (2.15–3.65)^***^	2.27 (1.71–3.00)^***^
75–84	1.80 (1.48–2.18)^***^	1.79 (1.47–2.17)^***^	1.56 (1.27–1.93)^***^
⩾85	1.31 (1.07–1.62)^**^	1.37 (1.11–1.69)^**^	1.20 (0.97–1.47)
Sub-acute phase stroke severity (NIHSS)			
0–4	2.83 (2.38–3.37)^***^	1.98 (1.66–2.36)^***^	1.84 (1.53–2.21)^***^
5–9	1.98 (1.52–2.58)^***^	1.80 (1.38–2.34)^***^	1.56 (1.18–2.11)^**^
⩾10	1.85 (1.47–2.34)	1.59 (1.26–2.00)^***^	1.32 (1.02–1.70)^*^
Premorbid disability (mRS)			
0–2	2.26 (1.94–2.64)^***^	1.68 (1.44–1.92)^***^	1.65 (1.40–1.94)^***^
⩾3	1.52 (1.22–1.89)^***^	1.56 (1.25–1.95)^***^	1.51 (1.20–1.90)^***^
Deprivation level (IMD)			
Most deprived^a^	2.58 (1.99–2.35)^***^	1.82 (1.40–2.38)^***^	1.59 (1.21–2.09)^**^
Least deprived^b^	2.43 (1.88–3.14)^***^	1.71 (1.32–2.22)^***^	1.49 (1.13–1.97)^**^
Smoking status			
Never smoked	2.37 (1.97–2.85)^***^	1.75 (1.45–2.10)^***^	1.38 (1.14–1.69)^**^
Ever smoked	2.50 (2.11–2.97)^***^	1.83 (1.54–2.17)^***^	1.77 (1.48–2.12)^***^
Depression or anxiety	2.89 (2.20–3.80)^***^	1.91 (1.44–2.52)^***^	1.80 (1.34–2.41)^***^
No depression or anxiety	2.29 (1.99–2.64)^***^	1.76 (1.53–2.03)^***^	1.53 (1.32–1.78)^***^
Hypertension	1.99 (1.70–2.32)^***^	1.70 (1.45–1.99)^***^	1.45 (1.24–1.71)^***^
No hypertension	3.08 (2.49–3.81)^***^	2.00 (1.61–2.49)^***^	1.78 (1.41–2.24)^***^
Hyperlipidemia	2.27 (1.77–2.90)^***^	1.85 (1.44–2.37)^***^	1.38 (1.06–1.79)^*^
No hyperlipidemia	2.55 (2.20–2.96)^***^	1.78 (1.53–2.07)^***^	1.58 (1.36–1.85)^***^
Atrial fibrillation	1.97 (1.54–2.50)^***^	1.91 (1.50–2.43)^***^	1.94 (1.50–2.51)^***^
No atrial fibrillation	2.22 (1.91–2.58)^***^	1.64 (1.41–1.91)^***^	1.52 (1.30–1.78)^***^

CI: confidence interval; CCI: Charlson comorbidity index; NIHSS: National Institutes of Health Stroke Scale; IMD: index of multiple deprivation; mRS: modified Rankin scale.

Expressed as hazard ratio (95% CI) for CCI ⩾ 2 versus 0. Model 3 included age, sex, and all other listed variables to the exception of the variable of interest.

* p < 0.05, ^**^p < 0.01, ^***^p < 0.001 between CCI groups.

a Highest quartile of IMD.

b Lowest quartile of IMD.

An analogous table using the weighted CCI is provided in the Supplemental Appendix.

Among the different etiological subtypes of ischemic stroke, CCI was most predictive in patients with small vessel disease stroke (10-year mortality-unweighted: aHR = 3.56, 2.14–5.91; weighted: 2.67, 1.70–4.21; both p < 0.001; Supplemental Appendix-Table S14 and Figure S10). Results were similar after limiting analyses to the low-risk THRIVE group (original or adjusted scoring; Supplemental Appendix-Table S15).

## Discussion

In this population-based study, pre-stroke multimorbidity was common and predictive of all-cause death at 1-, 5-, and 10 years post-stroke, driven particularly by non-vascular death. Multimorbidity was crudely associated with age, stroke severity, premorbid disability, deprivation, smoking, and other vascular risk factors (hypertension, hyperlipidemia, atrial fibrillation), but none of these potential confounders fully explained associations between multimorbidity and mortality. We also showed associations were strongest and most enduring in patients predicted to have a good initial outcome using the THRIVE score, and hence in younger patients, those without premorbid disability, and in minor or lacunar stroke.

Few previous studies have explored determinants of the predictive value of multimorbidity on mortality after stroke. In a recent scoping review,^2^ of seven studies relating multimorbidity to post-stroke mortality, none presented age-specific estimates or examined confounding by premorbid disability. Although multimorbidity and disability are independent constructs,^25,26^ multimorbidity has consistently been associated with greater disability in general population studies,^27–29^ such that mortality associations are often adjusted for premorbid disability,^28^ but to the best of our knowledge, no previous studies of post-stroke mortality have done so.^2^

Similarly, although previous studies on multimorbidity and mortality after stroke adjusted for NIHSS,^13,30^ none stratified by NIHSS or by etiological subtype. Since initial stroke severity is a key driver of early death post-stroke, predictive value of pre-stroke multimorbidity for later death after major stroke will inevitably be more limited by ceiling effects than after minor stroke. This same logic likely explains the lower predictive power of multimorbidity after strokes of unknown etiology (i.e. incomplete investigation generally for compassionate reasons due to poor prognosis) and the greater predictive power after lacunar stroke.

Among other potential confounders not included in previous studies of multimorbidity and post-stroke mortality, adjustment for deprivation, smoking, and anxiety/depression had minimal influence on observed associations, as was the case for comorbidities not included in the CCI (hypertension, hyperlipidemia, atrial fibrillation). In addition, to the best of our knowledge, our study is the first to calculate multimorbidity with ascertainment and follow-up completed using multiple methods including patient interviews and medical records as opposed to strictly secondary coding-based approaches used in previous studies in stroke.^2^

Our findings have some implications for clinicians and stroke services. First, case-mix adjustment in analyses of medium- and long-term outcomes after stroke should adjust for potential differences in multimorbidity. Although most comparisons of stroke services focus on short-term outcomes, longer-term outcome is also important, particularly in assessing the additional effects of rehabilitation and recurrent events. In a systematic review of case-mix adjustment models in stroke, none of the identified models included multimorbidity.^31^ Second, prognostic models for mortality after stroke should also include measures of multimorbidity. Knowledge of likely short- to medium-term mortality can have important implications for clinical decision-making, and also for key decisions patients and families have to make about issues, such as living arrangements, sale of property, and power of attorney. Third, when weighing up the potential risks and longer-term benefits of interventions to prevent recurrent events, an understanding of the likely risk of non-vascular death can potentially be helpful in aiding decisions whereby interventions might be futile. Previous studies have focused only on associations with all-cause death after stroke, which is of limited use in informing decisions to withhold interventions for secondary prevention as high overall mortality might reflect high rates of potentially preventable vascular deaths. We show the CCI more strongly predicts non-vascular mortality, largely driven by comorbidities, such as cancer and chronic obstructive pulmonary disease (COPD), and might therefore have some value identifying patients unlikely to survive long enough to benefit from interventions, such as patent foramen ovale (PFO) closure or endarterectomy for asymptomatic or moderate symptomatic carotid stenosis. Finally, there are currently no interventions specifically designed for stroke patients with multimorbidity,^2^ whereas multimorbidity-specific programs in the general population have shown modest success improving medication adherence, health-related behaviors, provider prescription patterns, and care quality.^32^ Interventions to improve long-term outcomes in stroke patients with multimorbidity may be justified.

Our study has several limitations. First, it is possible we under-ascertained comorbidities, although we used both patient interviews and hand-searched primary care records to minimize this rather than relying on routine electronic coding. Second, classification of cause of death as vascular versus non-vascular is sometimes subjective, particularly in relation to delayed complications of stroke. However, our coding was at least blind to multimorbidity status. Third, we used a version of the CCI that is limited to 15 conditions and does not include psychiatric disorders.^13^ However, it is the most widely used multimorbidity index in stroke research,^2^ and we found it predicted post-stroke mortality after adjustment for multiple non-CCI comorbidities and risk factors. Furthermore, in a preliminary comparison, we have found that the CCI is strongly correlated to the more detailed Elixhauser Index (Supplemental Appendix-Figure S11). Fourth, we did not have data available on pre-stroke frailty or cognitive status, which may have added additional nuance to our analyses, and further work should explore the potential interconnected links between pre-stroke multimorbidity, frailty, and cognition on outcome after stroke. However, previous work has highlighted how multimorbidity and frailty are different constructs,^25,26^ and the CCI includes history of dementia. Fifth, the mRS is scored subjectively and was designed to measure post-stroke functional status as opposed to premorbid status.^33^ However, the use of the mRS to measure premorbid functional status is common in stroke research and has been found to have concurrent validity with other measures of premorbid function, while being a strong prognostic factor for worse outcome following stroke.^33^ Finally, it is possible that patients with multimorbidity were less likely to receive acute treatments, less able to participate in rehabilitation after stroke, and were contraindicated for medications due to one or multiple pre-existing comorbidities. This may have biased associations between multimorbidity and vascular versus non-vascular death. While the intensity of care received in patients with pre-existing multimorbidity should be investigated in future work, our previous study that found no independent association between multimorbidity and severity of stroke reported sensitivity analyses whereby exclusion of patients who received thrombolysis or thrombectomy did not alter the results.^34^

In summary, pre-stroke multimorbidity is highly prevalent and an independent predictor of death after stroke, supporting its inclusion in models for case-mix adjustment and in informing decision-making for patients, families, and other carers. Multimorbidity was most predictive in younger patients, and after minor stroke, particularly for non-vascular death, suggesting potential utility in decision-making about interventions that require survival for 5–10 years to achieve a favorable risk/benefit ratio.

## Supplemental Material

sj-docx-1-wso-10.1177_17474930231210397 – Supplemental material for Association of multimorbidity with mortality after stroke stratified by age, severity, etiology, and prior disability Supplemental material, sj-docx-1-wso-10.1177_17474930231210397 for Association of multimorbidity with mortality after stroke stratified by age, severity, etiology, and prior disability by Matthew B Downer, Ramon Luengo-Fernandez, Lucy E Binney, Sergei Gutnikov, Louise E Silver, Aubretia McColl and Peter M Rothwell in International Journal of Stroke
