# Supplementary material for: Association of multimorbidity with mortality after stroke stratified by age, severity, etiology, and prior disability
Source: Int J Stroke. 2023 Nov 22;19(3):348–58. doi: 10.1177/17474930231210397 (PMC10903144; doi:10.1177/17474930231210397)
Supplement: sj-docx-1-wso-10.1177_17474930231210397 – Supplemental material for Association of multimorbidity with mortality after stroke stratified by age, severity, etiology, and prior disability [file sj-docx-1-wso-10.1177_17474930231210397.docx]

***Supplementary Material***

**Association of multimorbidity with mortality after stroke stratified by age, severity, aetiology, and prior disability**

Matthew B Downer MSc^1^, Ramon Luengo-Fernandez DPhil^1^, Lucy E Binney DM^1^, Sergei Gutnikov DPhil^1^, Louise E Silver DPhil^1^, Aubretia McColl DPhil^1^, Peter M Rothwell PhD FMedSci^1^, on behalf of the Oxford Vascular Study

^1^Wolfson Centre for the Prevention of Stroke and Dementia

Nuffield Department of Clinical Neurosciences

Wolfson Building - John Radcliffe Hospital

University of Oxford

Oxford, UK OX3 9DU

Correspondence:

Professor Peter M Rothwell

Email: peter.rothwell@ndcn.ox.ac.uk

Wolfson Centre for the Prevention of Stroke and Dementia

Nuffield Department of Clinical Neurosciences

Wolfson Building - John Radcliffe Hospital

University of Oxford. Oxford, UK OX3 9DU

**sTable 1:** Prevalence of Charlson Index comorbidities in the present sample of 2454 first-in-study period stroke patients

| **Comorbidity** | **Prevalence (N (%))** | **Weight/Points** |
| --- | --- | --- |
| AIDS/HIV | 2 (0.1) | 6 |
| Cancer (Solid Tumour) | 339 (13.8) | 2 |
| Cancer (Metastatic) | 26 (1.1) | 6 |
| Chronic Kidney Disease/Kidney Failure | 220 (8.9) | 2 |
| Chronic Obstructive Pulmonary Disease | 168 (6.9) | 1 |
| Congestive Heart Failure | 252 (10.2) | 1 |
| Connective Tissue Disease | 119 (4.9) | 1 |
| Dementia | 209 (8.5) | 1 |
| Diabetes (No End-Organ Damage) | 280 (11.4) | 1 |
| Diabetes (With End-Organ Damage) | 76 (3.1) | 2 |
| Leukaemia | 11 (0.5) | 2 |
| Liver Disease | 38 (1.6) | 1 |
| Lymphoma | 20 (0.8) | 2 |
| Myocardial Infarction | 280 (11.2) | 1 |
| Peptic Ulcer Disease | 199 (8.1) | 1 |
| Peripheral Vascular Disease | 236 (9.6) | 1 |

Weights used for the weighted version of the Charlson Comorbidity Index based off Goldstein et al. (2004)^1^. Note that both previous hemiplegia and stroke are not included.

**sTable 2:** Baseline clinical and demographic variables (using weighted Charlson Index groups)

| **Clinical/Demographic Variables** | **Charlson Comorbidity Index (Weighted)** | | | **Full Cohort (n=2454)** |
| --- | --- | --- | --- | --- |
|  | **0 (n=1079)** | **1 (n=477)** | **≥ 2 (n=898)** |  |
| Age*** | 71.00 (15.651) | 74.82 (13.16) | 78.30 (10.70) | 74.41 (13.92) |
| Male Sex | 537 (49.8%) | 236 (49.5%) | 428 (47.7%) | 1,201 (48.9%) |
| Pre-morbid mRS*** | 1 (0-1) | 1 (0-3) | 1 (1-3) | 1 (0-2) |
| Index of Multiple Deprivation** | 9.30 (5.89-13.06) | 9.83 (5.97-14.02) | 9.67 (5.78-13.76) | 9.67 (5.78-13.75) |
| Smoking History*** |  |  |  |  |
| *Non-Smoker* | 195 (18.1%) | 77 (16.1%) | 108 (12.0%) | 380 (15.5%) |
| *Ex-Smoker* | 374 (34.7%) | 202 (42.3%) | 395 (44.0%) | 971 (39.6%) |
| *Current Smoker* | 510 (47.3%) | 198 (41.5%) | 395 (44.0%) | 1,103 (44.9%) |
| History of Depression or Anxiety | 240 (22.2%) | 117 (24.5%) | 209 (23.3%) | 566 (23.1%) |
| Hypertension*** | 555 (51.4%) | 299 (62.7%) | 622 (69.3%) | 1,476 (60.1%) |
| Hyperlipidaemia*** | 201 (18.6%) | 144 (30.2%) | 297 (33.1%) | 642 (26.2%) |
| Atrial Fibrillation*** | 156 (14.5%) | 87 (18.2%) | 278 (31.0%) | 521 (21.2%) |
| Sub-Acute NIH Stroke Scale*** | 2 (1-7) | 3 (1-9) | 3 (1-9) | 3 (1-8) |
| Aetiological Subtype*** |  |  |  |  |
| *Intracerebral Haemorrhage* | 107 (9.9%) | 44 (9.2%) | 70 (7.8%) | 221 (9.0%) |
| *Subarachnoid Haemorrhage* | 56 (5.2%) | 15 (3.1%) | 24 (2.7%) | 95 (3.9%) |
| *Cardioembolic* | 227 (21.0%) | 121 (25.4%) | 296 (33.0%) | 644 (26.2%) |
| *Large Artery Disease* | 92 (8.5%) | 41 (8.6%) | 82 (9.1%) | 215 (8.8%) |
| *Small Vessel Disease* | 150 (13.9%) | 57 (11.9%) | 69 (7.7%) | 276 (11.2%) |
| *Undetermined* | 280 (25.9%) | 95 (19.9%) | 147 (16.4%) | 522 (21.3%) |
| *Unknown* | 108 (10.0%) | 77 (16.1%) | 147 (16.4%) | 332 (13.5%) |
| *Multiple* | 31 (2.9%) | 21 (4.4%) | 43 (4.8%) | 95 (3.9%) |
| *Other* | 28 (2.6%) | 6 (1.3%) | 20 (2.2%) | 54 (2.2%) |

**p<0.05, **p<0.01, ***p<0.001 between CCI groups (weighted)*

*CCI: Charlson Comorbidity Index, NIH: National Institutes of Health, mRS: Modified Rankin Scale*

*Data expressed as either count (percentage), mean (standard deviation), or median (interquartile range)*

**sTable 3:** Univariate and adjusted associations between pre-stroke multimorbidity and various pre-morbid/baseline variables (using the weighted Charlson Comorbidity Index)

| **Pre-morbid or Baseline Characteristic** | ***Odds Ratio (95%CI) for CCI ≥ 2 vs 0 (weighted/points)*** | | |
| --- | --- | --- | --- |
|  | **Model 1** | **Model 2** | **Model 3** |
|  | ***Crude*** | ***+ Age/Sex*** | ***+ All^3^*** |
| **Age^1^** |  |  |  |
| *<75* | 1.00 | 1.00 | 1.00 |
| *75-84* | 2.41 (1.96-2.97)*** | 2.43 (1.97-3.01)*** | 1.78 (1.42-2.24)*** |
| *≥ 85* | 2.54 (2.01-3.20)*** | 2.59 (2.04-3.28)*** | 1.59 (1.21-2.10)*** |
| **Sub-Acute Phase NIHSS^2^** |  |  |  |
| *5-9 vs 0-4* | 1.33 (1.04-1.71)* | 1.11 (0.85-1.43) | 1.04 (0.80-1.36) |
| *≥ 10 vs 0-4* | 1.44 (1.15-1.80)** | 1.18 (0.94-1.49) | 1.00 (0.79-1.28) |
| **Pre-morbid Disability (mRS)^2^** |  |  |  |
| *0-2* | 1.00 | 1.00 | 1.00 |
| *≥3* | 2.76 (2.22-3.45)*** | 2.17 (1.71-2.75)*** | 2.15 (1.68-2.76)*** |
| **Deprivation Level^1^** |  |  |  |
| *IMD Highest Quartile* | 1.00 | 1.00 | 1.00 |
| *IMD Lowest Quartile* | 1.11 (0.86-1.42) | 1.21 (0.93-1.57) | 1.11 (0.84-1.46) |
| **Smoking Status^1^** |  |  |  |
| *Never Smoked* | 1.00 | 1.00 | 1.00 |
| *Ever Smoked* | 1.14 (0.95-1.36) | 1.27 (1.05-1.55)* | 1.22 (1.00-1.50) |
| **Depression or Anxiety^2^** | 1.06 (0.86-1.31) | 1.17 (0.94-1.46) | 1.04 (0.82-1.32) |
| **Hypertension^2^** | 2.13 (1.77-2.56)*** | 1.77 (1.46-2.14)*** | 1.51 (1.23-1.85)*** |
| **Hyperlipidaemia^2^** | 2.16 (1.76-2.65)*** | 2.30 (1.85-2.85)*** | 2.12 (1.69-2.66)*** |
| **Atrial Fibrillation^2^** | 2.65 (2.13-3.31)*** | 2.08 (1.65-2.61)*** | 1.93 (1.51-2.46)*** |

*Variables denoted with a ^1^ were used as the predictor variable/Variables denoted as ^2^ were the outcome variable in the respective logistic regressions.*

*Expressed as odds ratio (95%CI) for CCI ≥ 2 vs CCI 0 (weighted)*

*Model 3 included age, sex, and all other listed variables to the exception of the variable of interest*

**p<0.05 **p<0.01 ***p<0.001 between CCI groups; CCI: Charlson Comorbidity Index, mRS: Modified Rankin Scale, NIHSS: National Institutes of Health Stroke Scale, IMD: Index of Multiple Deprivation*

**sTable 4:** Univariate and adjusted associations between pre-stroke multimorbidity and all-cause, vascular, and non-vascular mortality at various follow-up intervals after stroke

|  |  |  | |  | |  | | |  | |  |  |
| --- | --- | --- | --- | --- | --- | --- | --- | --- | --- | --- | --- | --- |
| **Model # / Adjustments** | | ***Hazard Ratios (95%CI) for CCI ≥ 2 vs 0 (weighted/points)*** | | | | | | | | | |  |
|  |  | **1 Year**  **All-Cause** | **5 Years**  **All-Cause** | | **10 Years**  **All-Cause** | | **10 Years**  **Vascular** | **10 Years**  **Non-Vascular** | |  |  |  |
| Model 1 | *Crude* | 1.92 (1.71-2.16) | 2.14 (1.90-2.41) | | 2.23 (1.98-2.51) | | 1.86 (1.55-2.22) | 2.54 (2.13-3.03) | | |  |  |
| Model 2 | *Age, Sex* | 1.48 (1.31-1.67) | 1.60 (1.42-1.81) | | 1.67 (1.48-1.88) | | 1.42 (1.18-1.70) | 1.88 (1.58-2.25) | | |  |  |
| Model 3A | *+ IMD* | 1.47 (1.31-1.66) | 1.60 (1.42-1.80) | | 1.66 (1.47-1.87) | | 1.41 (1.18-1.69) | 1.88 (1.58-2.25) | | |  |  |
| Model 3B | *+ Smoking* | 1.48 (1.31-1.67) | 1.61 (1.42-1.81) | | 1.66 (1.48-1.88) | | 1.42 (1.19-1.70) | 1.88 (1.57-2.24) | | |  |  |
| Model 3C | *+ Depression/Anxiety* | 1.49 (1.32-1.68) | 1.61 (1.43-1.81) | | 1.67 (1.48-1.88) | | 1.43 (1.19-1.71) | 1.88 (1.60-2.25) | | |  |  |
| Model 3D | *+ Prior Hypertension* | 1.48 (1.31-1.67) | 1.60 (1.42-1.81) | | 1.67 (1.48-1.89) | | 1.40 (1.17-1.68) | 1.94 (1.62-2.31) | | |  |  |
| Model 3E | *+ Prior Hyperlipidaemia* | 1.47 (1.31-1.66) | 1.61 (1.43-1.82) | | 1.68 (1.49-1.90) | | 1.40 (1.17-1.68) | 1.95 (1.63-2.33) | | |  |  |
| Model 3F | *+ Prior Atrial Fibrillation* | 1.43 (1.27-1.61) | 1.55 (1.38-1.75) | | 1.62 (1.43-1.82) | | 1.35 (1.13-1.62)^1^ | 1.85 (1.55-2.21) | | |  |  |
| Model 3G | *+ NIHSS* | 1.45 (1.29-1.63) | 1.59 (1.41-1.80) | | 1.65 (1.46-1.86) | | 1.35 (1.12-1.62)^1^ | 1.90 (1.59-2.26) | | |  |  |
| Model 3H | *+ mRS* | 1.39 (1.23-1.57) | 1.49 (1.32-1.68) | | 1.55 (1.38-1.75) | | 1.31 (1.09-1.57)^2^ | 1.77 (1.48-2.11) | | |  |  |
| Model 4 | *+ All* | 1.33 (1.17-1.50) | 1.44 (1.28-1.63) | | 1.51 (1.33-1.71) | | 1.18 (0.98-1.42)^3^ | 1.84 (1.53-2.21) | | |  |  |
|  |  |  | |  | |  | | |  | |  |  |

*Expressed as hazard ratio (95%CI) for CCI ≥ 2 vs 0 (weighted)*

*All p<0.001, except association denoted with ^1^(p=0.001) ^2^(p=0.004) ^3^(p=0.09)*

*CCI: Charlson Comorbidity Index, IMD: Index of Multiple Deprivation, NIHSS: National Institutes of Health Stroke Scale, mRS: Modified Rankin Scale*

**sTable 5:** Univariate and adjusted associations between pre-stroke multimorbidity and all-cause, vascular, and non-vascular mortality at various follow-up intervals after stroke (CCI included in the model as a continuous variable)

|  |  |  |  | |  |  |  |
| --- | --- | --- | --- | --- | --- | --- | --- |
| **Model # / Adjustments** | | ***HR (95%CI) per CCI comorbidity*** | | ***Hazard Ratio (95%I) per CCI point*** | | | |
|  |  | **10 Years; All-Cause Death** | | **10 Years; All-Cause Death** | | | |
| Model 1 | *Crude* | 1.21 (1.18-1.24) | | 1.33 (1.28-1.38) | | |  |
| Model 2 | *Age, Sex* | 1.14 (1.11-1.18) | | 1.22 (1.17-1.27) | | | |
| Model 3A | *+ IMD* | 1.14 (1.11-1.17) | | 1.22 (1.17-1.27) | | | |
| Model 3B | *+ Smoking* | 1.14 (1.11-1.18) | | 1.22 (1.17-1.27) | | | |
| Model 3C | *+ Depression/Anxiety* | 1.14 (1.11-1.18) | | 1.22 (1.17-1.27) | | | |
| Model 3D | *+ Prior Hypertension* | 1.14 (1.11-1.18) | | 1.22 (1.17-1.27) | | | |
| Model 3E | *+ Prior Hyperlipidaemia* | 1.14 (1.11-1.18) | | 1.22 (1.18-1.28) | | | |
| Model 3F | *+ Prior Atrial Fibrillation* | 1.13 (1.10-1.16) | | 1.20 (1.15-1.25) | | | |
| Model 3G | *+ NIHSS* | 1.14 (1.11-1.18) | | 1.23 (1.18-1.29) | | | |
| Model 3H | *+ mRS* | 1.12 (1.09-1.15) | | 1.17 (1.12-1.22) | | | |
| Model 4 | *+ All* | 1.11 (1.08-1.15) | | 1.18 (1.13-1.23) | | | |
|  |  |  |  | |  |  |  |

*Expressed as hazard ratio (95%CI) per CCI comorbidity or weighted CCI point; all p<0.001*

*CCI: Charlson Comorbidity Index, IMD: Index of Multiple Deprivation, NIHSS: National Institutes of Health Stroke Scale, mRS: Modified Rankin Scale*

**sTable 6:** Univariate and adjusted associations between pre-stroke multimorbidity and vascular and non-vascular mortality at 1 and 5-years follow-up after stroke

| **Model # / Adjustments** | | **Hazard Ratios (95%CI) for** **CCI ≥ 2 vs 0 (unweighted/count)** | | | |
| --- | --- | --- | --- | --- | --- |
|  |  | **1 Year** | | **5 Years** | |
|  |  | **Vascular** | **Non-Vascular** | **Vascular** | **Non-Vascular** |
| Model 1 | Crude | 1.65 (1.32-2.07)*** | 3.26 (2.34-4.56)*** | 1.83 (1.50-2.23)*** | 2.63 (2.11-3.29)*** |
| Model 2 | Age, Sex | 1.30 (1.04-1.64)* | 2.49 (1.77-3.49)*** | 1.40 (1.14-1.71)** | 1.91 (1.53-2.39)*** |
| Model 3A | + IMD | 1.29 (1.02-1.62)* | 2.48 (1.76-3.47)*** | 1.38 (1.13-1.69)** | 1.90 (1.52-2.38)*** |
| Model 3B | + Smoking | 1.32 (1.05-1.66)* | 2.52 (1.80-3.54)*** | 1.41 (1.15-1.73)** | 1.92 (1.53-2.41)*** |
| Model 3C | + Depression/Anxiety | 1.32 (1.05-1.67)* | 2.50 (1.78-3.50)*** | 1.42 (1.16-1.73)** | 1.92 (1.54-2.41)*** |
| Model 3D | + Prior Hypertension | 1.30 (1.03-1.64)* | 2.62 (1.86-3.69)*** | 1.38 (1.13-1.69)** | 1.96 (1.56-2.46)*** |
| Model 3E | + Prior Hyperlipidaemia | 1.32 (1.05-1.67)* | 2.68 (1.90-3.78)*** | 1.39 (1.14-1.71)** | 2.00 (1.59-2.51)*** |
| Model 3F | + Prior Atrial Fibrillation | 1.20 (0.95-1.51) | 2.35 (1.67-3.30)*** | 1.31 (1.07-1.61)** | 1.84 (1.47-2.31)*** |
| Model 3G | + NIHSS | 1.19 (0.95-1.50) | 2.42 (1.73-3.40)*** | 1.34 (1.09-1.63)** | 1.92 (1.53-2.40)*** |
| Model 3H | + mRS | 1.12 (0.89-1.41) | 2.14 (1.52-3.00)*** | 1.22 (1.00-1.50)* | 1.71 (1.36-2.14)*** |
| Model 4 | + All | 1.03 (0.80-1.31) | 2.30 (1.61-3.27)*** | 1.11 (0.90-1.37) | 1.78 (1.41-2.26)*** |

*Expressed as hazard ratio (95%CI) for CCI ≥ 2 vs 0 (unweighted/count)*

*CCI: Charlson Comorbidity Index, IMD: Index of Multiple Deprivation, NIHSS: National Institutes of Health Stroke Scale, mRS: Modified Rankin Scale*

**sTable 7:** Univariate and adjusted hazard ratios for all-cause death at 10 years post-stroke, stratified by pre-morbid/baseline variables

| **Pre-morbid or Baseline Characteristic** | ***Hazard Ratio (95%CI) for CCI ≥ 2 vs 0 (weighted/points)*** | | |
| --- | --- | --- | --- |
|  | **Model 1** | **Model 2** | **Model 3** |
|  | ***Crude*** | ***+ Age/Sex*** | ***+ All^3^*** |
| **Age (years)** |  |  |  |
| *< 75* | 3.20 (2.51-4.09)*** | 2.61 (2.03-3.35)*** | 2.28 (1.76-2.97)*** |
| *75-84* | 1.66 (1.39-2.00)*** | 1.65 (1.37-1.99)*** | 1.49 (1.22-1.80)*** |
| *≥ 85* | 1.24 (1.01-1.51)* | 1.28 (1.05-1.56)* | 1.15 (0.94-1.41) |
| **Sub-Acute Phase Stroke Severity (NIHSS)** |  |  |  |
| *0-4* | 2.51 (2.12-2.96)*** | 1.77 (1.50-2.10)*** | 1.72 (1.45-2.05)*** |
| *5-9* | 1.83 (1.42-2.35)*** | 1.67 (1.29-2.14)*** | 1.49 (1.14-1.95)** |
| *≥ 10* | 1.85 (1.49-2.31)*** | 1.57 (1.26-1.95)*** | 1.38 (1.09-1.74)** |
| **Pre-morbid Disability (mRS)** |  |  |  |
| *0-2* | 2.10 (1.82-2.42)*** | 1.54 (1.33-1.78)*** | 1.51 (1.30-1.76)*** |
| *≥ 3* | 1.58 (1.27-1.96)*** | 1.63 (1.31-2.02)*** | 1.56 (1.25-1.95)*** |
| **Deprivation Level (IMD)** |  |  |  |
| *Most Deprived^1^* | 2.32 (1.82-2.96)*** | 1.73 (1.35-2.21)*** | 1.54 (1.19-1.98)** |
| *Least Deprived^2^* | 2.26 (1.76-2.90)*** | 1.61 (1.25-2.08)*** | 1.41 (1.08-1.85)* |
| **Smoking Status** |  |  |  |
| *Never Smoked* | 2.13 (1.79-2.53)*** | 1.57 (1.32-1.86)*** | 1.32 (1.11-1.59)** |
| *Ever Smoked* | 2.35 (2.00-2.77)*** | 1.75 (1.49-2.07)*** | 1.74 (1.47-2.06)*** |
| **Depression or Anxiety** | 2.78 (2.13-3.61)*** | 1.87 (1.43-2.45)*** | 1.88 (1.42-2.49)*** |
| ***No Depression or Anxiety*** | 2.11 (1.84-2.41)*** | 1.61 (1.41-1.84)*** | 1.45 (1.26-1.67)*** |
| **Hypertension** | 1.92 (1.65-2.23)*** | 1.62 (1.40-1.89)*** | 1.44 (1.23-1.68)*** |
| ***No Hypertension*** | 2.60 (2.12-3.17)*** | 1.73 (1.41-2.12)*** | 1.61 (1.31-1.99)*** |
| **Hyperlipidaemia** | 2.14 (1.68-2.73)*** | 1.75 (1.37-2.24)*** | 1.35 (1.04-1.74)** |
| ***No Hyperlipidaemia*** | 2.31 (2.01-2.65)*** | 1.64 (1.43-1.89)*** | 1.54 (1.33-1.77)*** |
| **Atrial Fibrillation** | 1.95 (1.54-2.47)*** | 1.87 (1.48-2.37)*** | 1.93 (1.50-2.47)*** |
| ***No Atrial Fibrillation*** | 2.06 (1.79-2.37)*** | 1.52 (1.32-1.75)*** | 1.44 (1.25-1.67)*** |

*Expressed as hazard ratio (95%CI) for CCI ≥ 2 vs CCI 0 (weighted or unweighted). Model 3 included age, sex, and all other listed variables to the exception of the variable of interest. *p<0.05 **p<0.01 ***p<0.001 between CCI groups; CCI: Charlson Comorbidity Index, mRS: Modified Rankin Scale, NIHSS: National Institutes of Health Stroke Scale, IMD: Index of Multiple Deprivation.^1^Highest quartile of IMD; ^2^Lowest quartile of IMD.*

**sTable 8 (Set 1)**: Univariate and adjusted associations between pre-stroke multimorbidity (unweighted CCI) and potential confounders, stratified by age or sex

| **Variable/**  **Sub-Group** | ***Odds Ratio (95%CI) for CCI ≥ 2 (unweighted) vs 0*** | | |
| --- | --- | --- | --- |
|  | **Model 1**  ***Crude*** | **Model 2**  ***+ Age, Sex*** | **Model 3**  ***+ All^3^*** |
| **Sub-Acute Phase NIHSS (≥5)^2^** |  |  |  |
| *Age < 75* | 1.43 (0.87-2.35) | 1.37 (0.82-2.29) | 1.28 (0.72-2.30) |
| *Age ≥ 75* | 0.99 (0.71-1.39) | 0.99 (0.71-1.38) | 0.89 (0.62-1.28) |
| *Male* | 1.61 (1.09-2.38)* | 1.30 (0.87-1.94) | 1.14 (0.74-1.78) |
| *Female* | 1.15 (0.79-1.68) | 0.96 (0.65-1.42) | 0.92 (0.65-1.42) |
| **Pre-morbid Disability (mRS≥3)^2^** |  |  |  |
| *Age < 75* | 5.58 (3.18-9.79)*** | 5.29 (2.96-9.44)*** | 4.75 (2.55-8.87)*** |
| *Age ≥ 75* | 2.22 (1.70-2.90)*** | 2.53 (1.90-3.36)*** | 2.46 (1.81-3.32)*** |
| *Male* | 4.12 (2.81-6.05)*** | 2.99 (2.02-4.43)*** | 3.42 (2.25-5.24)*** |
| *Female* | 3.18 (2.36-4.30)*** | 2.66 (1.92-3.69)*** | 2.44 (1.72-3.47)*** |
| **Highest Deprivation Quartile^1^** |  |  |  |
| *Age < 75* | 1.57 (1.07-2.31)* | 1.69 (1.13-2.52)* | 1.63 (1.05-2.53)* |
| *Age ≥ 75* | 1.29 (0.95-1.77) | 1.32 (0.97-1.82) | 1.18 (0.83-1.66) |
| *Male* | 1.33 (0.96-1.85) | 1.47 (1.04-2.08)* | 1.33 (0.91-1.95) |
| *Female* | 1.33 (0.95-1.87) | 1.34 (0.94-1.89) | 1.26 (0.86-1.84) |
| **History of Smoking^1^** |  |  |  |
| *Age < 75* | 1.32 (0.95-1.83) | 1.29 (0.91-1.83) | 1.24 (0.85-1.83) |
| *Age ≥ 75* | 1.48 (1.15-1.91)** | 1.38 (1.06-1.80)* | 1.35 (1.01-1.80)* |
| *Male* | 1.40 (1.04-1.88)* | 1.35 (0.99-1.85) | 1.21 (0.86-1.70) |
| *Female* | 1.21 (0.92-1.59) | 1.38 (1.03-1.83)* | 1.44 (1.06-1.97)* |
| **Previous Depression or Anxiety^2^** |  |  |  |
| *Age < 75* | 1.08 (0.75-1.55) | 1.27 (0.87-1.84) | 1.07 (0.71-1.62) |
| *Age ≥ 75* | 1.29 (0.95-1.75) | 1.37 (1.01-1.87)* | 1.21 (0.87-1.68) |
| *Male* | 0.85 (0.59-1.22) | 1.03 (0.71-1.50) | 0.91 (0.61-1.37) |
| *Female* | 1.42 (1.05-1.91)* | 1.54 (1.13-2.08)** | 1.31 (0.94-1.83) |
| **Prior Hypertension^2^** |  |  |  |
| *Age < 75* | 3.04 (2.17-4.26)*** | 2.35 (1.66-3.34)*** | 1.86 (1.28-2.70)** |
| *Age ≥ 75* | 1.59 (1.21-2.08)** | 1.64 (1.25-2.14)*** | 1.45 (1.08-1.94)* |
| *Male* | 2.12 (1.60-2.81)*** | 1.69 (1.26-2.27)*** | 1.29 (0.93-1.78) |
| *Female* | 2.52 (1.87-3.38)*** | 2.16 (1.59-2.92)*** | 2.02 (1.44-2.83)*** |
| **Prior Hyperlipidaemia^2^** |  |  |  |
| *Age < 75* | 3.49 (2.48-4.92)*** | 3.13 (2.20-4.44)*** | 2.82 (1.93-4.13)*** |
| *Age ≥ 75* | 2.21 (1.65-2.98)*** | 2.31 (1.71-3.11)*** | 2.20 (1.59-3.03)*** |
| *Male* | 3.21 (2.36-4.37)*** | 3.45 (2.49-4.78)*** | 3.71 (2.61-5.38)*** |
| *Female* | 2.02 (1.48-2.76)*** | 2.17 (1.58-2.98)*** | 1.83 (1.30-2.58)*** |
| **Prior Atrial Fibrillation^2^** |  |  |  |
| *Age < 75* | 3.16 (2.01-4.97)*** | 2.44 (1.53-3.88)*** | 1.95 (1.16-3.30)* |
| *Age ≥ 75* | 2.42 (1.83-3.20)*** | 2.47 (1.86-3.28)*** | 2.37 (1.75-3.22)*** |
| *Male* | 3.21 (2.29-4.51)*** | 2.34 (1.64-3.32)*** | 2.01 (1.37-2.96)*** |
| *Female* | 3.08 (2.24-4.24)*** | 2.57 (1.85-3.58)*** | 2.57 (1.79-3.69)*** |

*^1^indicates the variable was included as the predictor variable in respective logistic regression model, ^2^indicates variable included as outcome variable.*

*All variables were associated with unweighted CCI (≥2 vs 0). All values indicate odds ratio (95%CI).*

*Model 3 included age, sex, and all other listed variables to the exception of the variable of interest*

**p<0.05 **p<0.001 between CCI groups; CCI: Charlson Comorbidity Index, mRS: Modified Rankin Scale.*

**sTable 8 (Set 2)**: Univariate and adjusted associations between pre-stroke multimorbidity (weighted CCI) and potential confounders, stratified by age or sex

| **Variable/Sub-Group** | ***Odds Ratio (95%CI) for CCI ≥ 2 (weighted) vs 0*** | | |
| --- | --- | --- | --- |
|  | **Model 1**  ***Crude*** | **Model 2**  ***+ Age, Sex*** | **Model 3**  ***+ All^3^*** |
| **Sub-Acute Phase NIHSS (≥5)^2^** |  |  |  |
| *Age < 75* | 1.59 (1.02-2.46)* | 1.44 (0.92-2.26) | 1.28 (0.78-2.08) |
| *Age ≥ 75* | 0.95 (0.70-1.31) | 0.95 (0.69-1.30) | 0.86 (0.61-1.21) |
| *Male* | 1.57 (1.08-2.27)* | 1.26 (0.86-1.85) | 1.15 (0.76-1.74) |
| *Female* | 1.15 (0.81-1.62) | 0.98 (0.69-1.40) | 0.96 (0.66-1.39) |
| **Pre-morbid Disability (mRS≥3)^2^** |  |  |  |
| *Age < 75* | 4.72 (2.74-8.12)*** | 4.42 (2.53-7.74)*** | 4.17 (2.31-7.54)*** |
| *Age ≥ 75* | 1.79 (1.39-2.32)*** | 1.95 (1.49-2.56)*** | 1.90 (1.43-2.52)*** |
| *Male* | 3.60 (2.48-5.23)*** | 2.60 (1.77-3.82)*** | 2.88 (1.92-4.33)*** |
| *Female* | 2.38 (1.80-3.16)*** | 1.96 (1.44-2.65)*** | 1.86 (1.35-2.57)*** |
| **Highest Deprivation Quartile^1^** |  |  |  |
| *Age < 75* | 1.19 (0.80-1.77) | 1.25 (0.83-1.90) | 1.15 (0.74-1.79) |
| *Age ≥ 75* | 1.20 (0.85-1.70) | 1.21 (0.86-1.71) | 1.11 (0.77-1.60) |
| *Male* | 1.26 (0.88-1.81) | 1.43 (0.98-2.11) | 1.31 (0.87-1.97) |
| *Female* | 0.96 (0.67-1.37) | 1.02 (0.71-1.48) | 0.96 (0.65-1.40) |
| **History of Smoking^1^** |  |  |  |
| *Age < 75* | 1.26 (0.94-1.69) | 1.29 (0.94-1.76) | 1.26 (0.90-1.76) |
| *Age ≥ 75* | 1.27 (1.00-1.60)* | 1.21 (0.94-1.55) | 1.15 (0.88-1.49) |
| *Male* | 1.29 (0.98-1.70) | 1.24 (0.93-1.66) | 1.11 (0.81-1.52) |
| *Female* | 1.09 (0.85-1.40) | 1.24 (0.96-1.61) | 1.25 (0.95-1.64) |
| **Previous Depression or Anxiety^2^** |  |  |  |
| *Age < 75* | 0.97 (0.70-1.35) | 1.10 (0.78-1.56) | 0.95 (0.65-1.38) |
| *Age ≥ 75* | 1.22 (0.92-1.63) | 1.27 (0.95-1.70) | 1.15 (0.85-1.56) |
| *Male* | 0.86 (0.62-1.20) | 1.04 (0.73-1.48) | 0.92 (0.63-1.34) |
| *Female* | 1.20 (0.91-1.59) | 1.29 (0.97-1.71) | 1.15 (0.85-1.56) |
| **Prior Hypertension^2^** |  |  |  |
| *Age < 75* | 2.45 (1.82-3.29)*** | 1.92 (1.41-2.61)*** | 1.56 (1.12-2.17)*** |
| *Age ≥ 75* | 1.59 (1.24-2.04)*** | 1.62 (1.26-2.08)*** | 1.49 (1.14-1.95)** |
| *Male* | 2.01 (1.55-2.61)*** | 1.60 (1.22-2.11)** | 1.26 (0.94-1.70) |
| *Female* | 2.24 (1.72-2.91)*** | 1.95 (1.49-2.55)*** | 1.90 (1.41-2.55)*** |
| **Prior Hyperlipidaemia^2^** |  |  |  |
| *Age < 75* | 2.86 (2.08-3.93)*** | 2.57 (1.86-3.56)*** | 2.38 (1.69-3.37)*** |
| *Age ≥ 75* | 1.89 (1.42-2.50)*** | 1.94 (1.45-2.58)*** | 1.79 (1.32-2.43)*** |
| *Male* | 2.81 (2.09-3.78)*** | 3.00 (2.20-4.11)*** | 3.14 (2.25-4.38)*** |
| *Female* | 1.67 (1.25-2.24)** | 1.78 (1.32-2.40)*** | 1.52 (1.11-2.08)** |
| **Prior Atrial Fibrillation^2^** |  |  |  |
| *Age < 75* | 2.65 (1.72-4.08)*** | 2.06 (1.32-3.21)** | 1.67 (1.02-2.73)* |
| *Age ≥ 75* | 2.06 (1.58-2.74)*** | 2.09 (1.59-2.74)*** | 1.96 (1.47-2.61)*** |
| *Male* | 2.86 (2.06-3.97)*** | 2.08 (1.48-2.92)*** | 1.78 (1.23-2.57)** |
| *Female* | 2.48 (1.83-3.34)*** | 2.08 (1.52-2.84)*** | 2.01 (1.44-2.81)*** |

*^1^indicates the variable was included as the predictor variable in respective logistic regression model, ^2^indicates variable included as outcome variable.*

*All variables were associated with weighted CCI (≥2 vs 0). All values indicate odds ratio (95%CI).*

*Model 3 included age, sex, and all other listed variables to the exception of the variable of interest*

**p<0.05 **p<0.001 between CCI groups; CCI: Charlson Comorbidity Index, mRS: Modified Rankin Scale.*

**sTable 9:** Univariate and adjusted associations between pre-stroke multimorbidity and all-cause mortality at 10 years follow-up, stratified by age or sex

| **Model # / Adjustments** | | ***A: Hazard Ratio (95%CI) for CCI ≥ 2 vs 0 (unweighted) for 10-year mortality*** | | | |
| --- | --- | --- | --- | --- | --- |
|  |  | **Age < 75** | **Age ≥ 75** | **Male** | **Female** |
| Model 1 | Crude | 3.46 (2.67-4.58)*** | 1.57 (1.36-1.80)*** | 2.94 (2.44-3.54)*** | 2.05 (1.73-2.42)*** |
| Model 2 | Age, Sex | 2.80 (2.15-3.65)*** | 1.58 (1.37-1.82)*** | 1.98 (1.64-2.38)*** | 1.65 (1.39-1.96)*** |
| Model 3A | + IMD | 2.78 (2.14-3.62)*** | 1.57 (1.36-1.81)*** | 1.96 (1.62-2.36)*** | 1.64 (1.38-1.95)*** |
| Model 3B | + Smoking | 2.83 (2.17-3.68)*** | 1.58 (1.37-1.82)*** | 1.97 (1.63-2.38)*** | 1.65 (1.39-1.96)*** |
| Model 3C | + Depression/Anxiety | 2.81 (2.16-3.66)*** | 1.58 (1.37-1.82)*** | 1.97 (1.63-2.37)*** | 1.65 (1.39-1.96)*** |
| Model 3D | + Hypertension | 2.87 (2.19-3.76)*** | 1.58 (1.37-1.82)*** | 1.99 (1.65-2.41)*** | 1.65 (1.39-1.97)*** |
| Model 3E | + Hyperlipidaemia | 2.87 (2.19-3.76)*** | 1.59 (1.38-1.84)*** | 2.09 (1.72-2.53)*** | 1.64 (1.38-1.95)*** |
| Model 3F | + Atrial Fibrillation | 2.72 (2.08-3.54)*** | 1.51 (1.31-1.74)*** | 1.90 (1.57-2.30)*** | 1.58 (1.33-1.87)*** |
| Model 3G | + NIHSS | 2.47 (1.89-3.23)*** | 1.61 (1.39-1.86)*** | 1.96 (1.62-2.37)*** | 1.65 (1.39-1.95)*** |
| Model 3H | + mRS | 2.32 (1.78-3.03)*** | 1.42 (1.23-1.64)*** | 1.81 (1.50-2.19)*** | 1.43 (1.20-1.70)*** |
| Model 4 | + All | 2.27 (1.71-3.00)*** | 1.39 (1.20-1.62)*** | 1.82 (1.49-2.22)*** | 1.36 (1.13-1.62)** |

| **Model # / Adjustments** | | ***B: Hazard Ratio (95%CI) for CCI ≥ 2 vs 0 (weighted) for 10-year mortality*** | | | |
| --- | --- | --- | --- | --- | --- |
|  |  | **Age < 75** | **Age ≥ 75** | **Male** | **Female** |
| Model 1 | Crude | 3.20 (2.51-4.09)*** | 1.46 (1.27-1.67)*** | 2.74 (2.29-3.28)*** | 1.87 (1.60-2.20)*** |
| Model 2 | Age, Sex | 2.61 (2.03-3.35)*** | 1.47 (1.28-1.68)*** | 1.84 (1.53-2.20)*** | 1.54 (1.31-1.81)*** |
| Model 3A | + IMD | 2.60 (2.03-3.34)*** | 1.46 (1.28-1.67)*** | 1.82 (1.52-2.19)*** | 1.54 (1.32-1.81)*** |
| Model 3B | + Smoking | 2.62 (2.04-3.37)*** | 1.46 (1.28-1.68)*** | 1.84 (1.53-2.20)*** | 1.54 (1.31-1.81)*** |
| Model 3C | + Depression/Anxiety | 2.61 (2.03-3.35)*** | 1.47 (1.28-1.68)*** | 1.83 (1.53-2.20)*** | 1.54 (1.32-1.81)*** |
| Model 3D | + Hypertension | 2.65 (2.06-3.41)*** | 1.46 (1.28-1.68)*** | 1.85 (1.54-2.22)*** | 1.54 (1.31-1.82)*** |
| Model 3E | + Hyperlipidaemia | 2.66 (2.06-3.43)*** | 1.47 (1.28-1.69)*** | 1.93 (1.60-2.32)*** | 1.53 (1.31-1.80)*** |
| Model 3F | + Atrial Fibrillation | 2.56 (1.99-3.28)*** | 1.42 (1.24-1.63)*** | 1.79 (1.49-2.15)*** | 1.49 (1.27-1.75)*** |
| Model 3G | + NIHSS | 2.42 (1.88-3.12)*** | 1.46 (1.28-1.68)*** | 1.86 (1.55-2.23)*** | 1.51 (1.28-1.77)*** |
| Model 3H | + mRS | 2.26 (1.76-2.91)*** | 1.38 (1.20-1.58)*** | 1.75 (1.45-2.09)*** | 1.41 (1.20-1.66)*** |
| Model 4 | + All | 2.28 (1.76-2.97)*** | 1.34 (1.16-1.54)*** | 1.78 (1.47-2.15)*** | 1.33 (1.13-1.57)** |

*Expressed as hazard ratio (95%CI) for CC ≥I 2 vs 0 (unweighted or weighted).*

***p<0.01 ***p<0.001*

*CCI: Charlson Comorbidity Index, IMD: Index of Multiple Deprivation, NIHSS: National Institutes of Health Stroke Scale, mRS: Modified Rankin Scale*

**sTable 10:** Univariate and adjusted associations between pre-stroke multimorbidity and all-cause mortality at 10 years follow-up, stratified by type of haemorrhagic stroke

| **Model # / Adjustments** | | ***HR (95%CI) for CCI ≥ 2 vs 0 (unweighted)*** | | ***HR (95%CI) for CCI ≥ 2 vs 0 (weighted)*** | |
| --- | --- | --- | --- | --- | --- |
|  |  | **ICH (n=221)** | **SAH (n=95)** | **ICH (n=221)** | **SAH (n=95)** |
| Model 1 | Crude | 1.68 (1.10-2.57)* | 1.98 (0.77-5.13)* | 1.74 (1.19-2.53)** | 1.52 (0.83-2.80) |
| Model 2 | Age, Sex | 1.25 (0.81-1.93) | 1.97 (0.88-4.37) | 1.26 (0.86-1.86) | 0.99 (0.52-1.88) |
| Model 3B | + IMD | 1.24 (0.80-1.91) | 2.08 (0.93-4.62) | 1.26 (0.85-1.86) | 0.97 (0.51-1.83) |
| Model 3C | + Smoking | 1.26 (0.82-1.93) | 1.98 (0.89-4.39) | 1.25 (0.85-1.85) | 0.99 (0.52-1.89) |
| Model 3D | + Depression/Anxiety | 1.26 (0.82-1.93) | 2.08 (0.92-4.70) | 1.26 (0.86-1.86) | 0.99 (0.52-1.90) |
| Model 3D | + Prior Hypertension | 1.20 (0.78-1.85) | 2.00 (0.90-4.47) | 1.21 (0.82-1.79) | 0.95 (0.50-1.83) |
| Model 3E | + Prior Hyperlipidaemia | 1.21 (0.78-1.87) | 1.88 (0.81-4.34) | 1.25 (0.85-1.84) | 0.92 (0.46-1.85) |
| Model 3F | + Prior Atrial Fibrillation | 1.24 (0.80-1.91) | 1.79 (0.79-4.03) | 1.25 (0.84-1.84) | 0.99 (0.53-1.86) |
| Model 3G | + NIH Stroke Scale | 1.02 (0.66-1.58) | 1.85 (0.82-4.19) | 1.08 (0.73-1.60) | 1.21 (0.63-2.31) |
| Model 3G | + mRS | 1.13 (0.73-1.75) | 1.91 (0.86-4.24) | 1.21 (0.82-1.79) | 0.95 (0.50-1.81) |
| Model 4 | + All | 0.97 (0.61-1.52) | 1.85 (0.73-4.67) | 1.07 (0.72-1.61) | 1.05 (0.52-2.10) |

*Expressed as hazard ratio (95%CI) for CC ≥I 2 vs 0 (unweighted or weighted).*

**p<0.05 **p<0.01*

*CCI: Charlson Comorbidity Index, IMD: Index of Multiple Deprivation, NIH: National Institutes of Health, mRS: Modified Rankin Scale.*

**sTable 11:** Univariate and adjusted associations between pre-stroke comorbidities in the Charlson Index and all-cause, vascular, and no-vascular mortality at 10 years follow-up

| **CCI Comorbidity** | **Vascular Mortality** | | **Non-Vascular Mortality** | | **All-Cause Mortality** | |
| --- | --- | --- | --- | --- | --- | --- |
|  | **Crude** | **Age/Sex Adjusted** | **Crude** | **Age/Sex Adjusted** | **Crude** | **Age/Sex Adjusted** |
| AIDS/HIV | N/A | N/A | N/A | N/A | N/A | N/A |
| Cancer (Solid Tumour) | 1.30 (1.05-1.61)* | 1.03 (0.83-1.28) | 1.77 (1.44-2.18)*** | 1.37 (1.11-1.68)** | 1.54 (1.34-1.77)*** | 1.19 (1.04-1.37)* |
| Cancer (Metastatic) | 1.16 (0.48-2.79) | 1.15 (0.47-2.77) | 6.93 (4.20-11.42)*** | 7.00 (4.24-11.55)*** | 3.34 (2.23-5.01)*** | 3.36 (2.24-5.04)*** |
| Chronic Kidney Disease/Kidney Failure | 1.21 (0.93-1.59) | 0.95 (0.72-1.24) | 1.93 (1.51-2.45)*** | 1.43 (1.12-1.82)** | 1.54 (1.30-1.82)*** | 1.16 (0.98-1.37) |
| Chronic Obstructive Pulmonary Disease | 1.10 (0.81-1.49) | 1.19 (0.88-1.62) | 1.83 (1.41-2.37)*** | 1.97 (1.52-2.56)*** | 1.43 (1.19-1.73)*** | 1.56 (1.30-1.89)*** |
| Congestive Heart Failure | 2.33 (1.90-2.87)*** | 1.74 (1.42-2.15)*** | 2.38 (1.91-2.99)*** | 1.77 (1.41-2.21)*** | 2.25 (1.95-2.61)*** | 1.66 (1.43-1.92)*** |
| Connective Tissue Disease | 1.12 (0.79-1.58) | 0.94 (0.67-1.33) | 1.31 (0.94-1.81) | 1.06 (0.76-1.48) | 1.16 (0.92-1.46) | 0.95 (0.76-1.20) |
| Dementia | 2.24 (1.76-2.86)*** | 1.34 (1.05-1.71)* | 3.08 (2.39-3.98)*** | 1.77 (1.36-2.29)*** | 2.88 (2.45-3.38)*** | 1.64 (1.39-1.93)*** |
| Diabetes | 1.18 (0.95-1.47) | 1.27 (1.03-1.58)* | 1.17 (0.94-1.46) | 1.24 (0.99-1.55) | 1.21 (1.05-1.39)* | 1.30 (1.12-1.50)*** |
| Liver Disease | 1.18 (0.65-2.14) | 2.00 (1.10-3.64)* | 1.11 (0.59-2.07) | 2.15 (1.15-4.04)* | 1.20 (0.81-1.79) | 2.27 (1.52-3.37)*** |
| Leukaemia | 1.88 (0.70-5.02) | 1.47 (0.55-3.92) | 1.75 (0.56-5.42) | 1.30 (0.42-4.06) | 1.64 (0.78-3.44) | 1.25 (0.59-2.63) |
| Lymphoma | 1.72 (0.71-3.17) | 1.51 (0.71-3.17) | 2.89 (1.50-5.87)** | 2.36 (1.22-4.57)* | 2.00 (1.22-3.28)** | 1.69 (1.03-2.76)* |
| Myocardial Infarction | 1.65 (1.33-2.03)*** | 1.44 (1.16-1.78)*** | 1.31 (1.04-1.66)* | 1.06 (0.84-1.35) | 1.49 (1.28-1.72)*** | 1.25 (1.08-1.45)** |
| Peptic Ulcer Disease | 1.10 (0.83-1.45) | 0.99 (.75-1.31) | 1.71 (1.34-2.17)*** | 1.42 (1.12-1.81)** | 1.39 (1.17-1.65)*** | 1.21 (1.02-1.43)* |
| Peripheral Vascular Disease | 1.62 (1.29-2.04)*** | 1.54 (1.22-1.95)*** | 1.62 (1.27-2.05)*** | 1.46 (1.15-1.85)** | 1.58 (1.35-1.84)*** | 1.46 (1.25-1.71)*** |

*Expressed as hazard ratio (95%CI)*

**p<0.05 **p<0.01***p<0.001*

*CCI: Charlson Comorbidity Index. N/A: No hazard ratios were obtained for HIV/AIDS, as only 2 patients had a diagnosis at the time of stroke.*

**sTable 12:** Univariate and adjusted associations between pre-stroke multimorbidity and all-cause mortality at 90-days (Set A), 1-year (Set B), and 10-years (Set C) follow-up, stratified by THRIVE Score Risk Groups

| **A** | **THRIVE Risk Group** | **Hazard Ratio (95%CI) for CCI ≥ 2 vs 0 (unweighted/counts)** | | **Hazard Ratio (95%CI) for CCI ≥ 2 vs 0 (weighted/points)** | |
| --- | --- | --- | --- | --- | --- |
|  |  | *Model 1 - Crude* | *Model 2 -*  *Adjusted for Age/Sex* | *Model 1 - Crude* | *Model 2 -*  *Adjusted for Age/Sex* |
| *Original Scoring* | Low (0-2) | 1.51 (0.93-2.45) | 1.27 (0.78-2.07) | 1.56 (1.03-2.36)* | 1.30 (0.85-1.97) |
|  | Moderate (3-5) | 1.18 (0.87-1.59) | 1.19 (0.88-1.61) | 1.20 (0.90-1.61) | 1.20 (0.90-1.61) |
|  | High (6-9) | 0.97 (0.64-1.48) | 0.99 (0.65-1.52) | 1.03 (0.69-1.54) | 1.07 (0.72-1.60) |
| *Adj. NIHSS* | Low (0-2) | 1.63 (0.78-3.42) | 1.45 (0.69-3.06) | 1.78 (0.96-3.32) | 1.55 (0.83-2.91) |
|  | Moderate (3-5) | 0.99 (0.69-1.40) | 0.98 (0.69-1.40) | 1.00 (0.71-1.40) | 1.00 (0.71-1.40) |
|  | High (6-9) | 1.15 (0.86-1.54) | 1.17 (0.86-1.57) | 1.21 (0.92-1.59) | 1.22 (0.93-1.62) |

| **B** | **THRIVE Risk Group** | **Hazard Ratio (95%CI) for CCI ≥ 2 vs 0 (unweighted/counts)** | | | **Hazard Ratio (95%CI) for CCI ≥ 2 vs 0 (weighted/points)** | |
| --- | --- | --- | --- | --- | --- | --- |
|  |  | *Model 1 -*  *Crude* | *Model 2 -*  *Adjusted for Age/Sex* | | *Model 1 -*  *Crude* | *Model 2 -*  *Adjusted for Age/Sex* |
| *Original Scoring* | Low (0-2) | 2.11 (1.45-3.08)*** | | 1.68 (1.15-2.47)** | 2.07 (1.48-2.88)*** | 1.65 (1.18-2.31)** |
|  | Moderate (3-5) | 1.38 (1.07-1.78)* | | 1.41 (1.09-1.82)** | 1.38 (1.08-1.76)* | 1.39 (1.08-1.77)** |
|  | High (6-9) | 0.94 (0.63-1.39) | | 0.96 (0.65-1.42) | 1.00 (0.69-1.44) | 1.03 (0.71-1.49) |
| *Adj. NIHSS* | Low (0-2) | 2.48 (1.49-4.12)*** | | 1.97 (1.17-3.30)* | 2.24 (1.12-2.84)** | 1.78 (1.12-2.83)* |
|  | Moderate (3-5) | 1.23 (0.92-1.65) | | 1.21 (0.90-1.62) | 1.25 (0.95-1.65) | 1.22 (0.93-1.61) |
|  | High (6-9) | 1.29 (0.99-1.67) | | 1.30 (1.00-1.70) | 1.33 (1.03-1.71)* | 1.34 (1.04-1.72)* |

| **C** | **THRIVE Risk Group** | **Hazard Ratio (95%CI) for CCI ≥ 2 vs 0 (unweighted/counts)** | | | **Hazard Ratio (95%CI) for CCI ≥ 2 vs 0 (weighted/points)** | |
| --- | --- | --- | --- | --- | --- | --- |
|  |  | *Model 1 -*  *Crude* | *Model 2 -*  *Adjusted for Age/Sex* | | *Model 1 -*  *Crude* | *Model 2 -*  *Adjusted for Age/Sex* |
| *Original Scoring* | Low (0-2) | 2.72 (2.16-3.41)*** | | 2.11 (1.67-2.66)*** | 2.23 (1.81-2.75)*** | 1.71 (1.39-2.10)*** |
|  | Moderate (3-5) | 1.40 (1.18-1.63)*** | | 1.49 (1.26-1.77)*** | 1.43 (1.2101.69)*** | 1.48 (1.26-1.75)*** |
|  | High (6-9) | 0.99 (0.69-1.42) | | 1.01 (0.70-1.46) | 1.00 (0.71-1.41) | 1.04 (0.73-1.47) |
| *Adj. NIHSS* | Low (0-2) | 2.95 (1.26-3.86)*** | | 2.22 (1.69-2.91)*** | 2.29 (1.79-2.93)*** | 1.69 (1.31-2.16)*** |
|  | Moderate (3-5) | 1.51 (1.26-1.82)*** | | 1.48 (1.23-1.78)*** | 1.53 (1.28-1.82)*** | 1.46 (1.22-1.74)*** |
|  | High (6-9) | 1.40 (1.11-1.76)** | | 1.41 (1.12-1.78)** | 1.43 (1.15-1.78)** | 1.43 (1.15-1.78)** |

*Note: Original THRIVE scoring includes the following NIHSS categories (NIHSS ≤ 10: 0 points, 11-20: 2 points, ≥ 21: 4 points) based on NIHSS on presentation. We also used THRIVE with adjusted NIHSS scoring (NIHSS ≤ 4: 0 points, 5-9: 2 points, ≥ 10: 4 points), as the present study included sub-acute phase NIHSS*

**sTable 13.** Absolute risks of all-cause death at 10 years post-stroke by Charlson Comorbidity Index group, stratified by age and sex

| **Age Group** | **Full Cohort** | | | **Males** | | | **Females** | | |
| --- | --- | --- | --- | --- | --- | --- | --- | --- | --- |
|  | **AR**  **CCI ≥ 2** | **AR**  **CCI = 0** | **ARD (95%CI)** | **AR**  **CCI ≥ 2** | **AR**  **CCI = 0** | **ARD (95%CI)** | **AR**  **CCI ≥ 2** | **AR**  **CCI = 0** | **ARD (95%CI)** |
| *Unweighted CCI* |  |  |  |  |  |  |  |  |  |
| All Ages | 0.76 | 0.45 | 0.31 (0.27-0.36) | 0.75 | 0.37 | 0.38 (0.32-0.44) | 0.77 | 0.52 | 0.25 (0.19-0.31) |
| Less Than 65 | 0.45 | 0.13 | 0.32 (0.20-0.43) | 0.42 | 0.11 | 0.31 (0.16-0.47) | 0.49 | 0.17 | 0.31 (0.14-0.49) |
| 65-74 | 0.60 | 0.30 | 0.31 (0.21-.0.41) | 0.63 | 0.27 | 0.36 (0.23-0.48) | 0.56 | 0.33 | 0.23 (0.07-0.40) |
| 75-84 | 0.83 | 0.62 | 0.20 (0.13-0.27) | 0.82 | 0.63 | 0.19 (0.09-0.30) | 0.83 | 0.62 | 0.21 (0.11-0.31) |
| Greater than 85 | 0.90 | 0.86 | 0.04 (-0.03-0.10) | 0.92 | 0.85 | 0.07 (-0.03-0.18) | 0.89 | 0.86 | 0.02 (-0.06-0.10) |
| *Weighted CCI* |  |  |  |  |  |  |  |  |  |
| All Ages | 0.72 | 0.45 | 0.28 (0.23-0.32) | 0.71 | 0.37 | 0.35 (0.29-0.40) | 0.73 | 0.52 | 0.21 (0.15-0.26) |
| Less Than 65 | 0.41 | 0.13 | 0.28 (0.18-0.38) | 0.40 | 0.11 | 0.30 (0.16-0.44) | 0.42 | 0.17 | 0.25 (0.10-0.39) |
| 65-74 | 0.58 | 0.30 | 0.28 (0.19-0.38) | 0.58 | 0.27 | 0.31 (0.20-0.43) | 0.58 | 0.33 | 0.25 (0.10-0.40) |
| 75-84 | 0.78 | 0.62 | 0.16 (0.09-0.23) | 0.79 | 0.63 | 0.16 (0.06-0.26) | 0.77 | 0.62 | 0.15 (0.06-0.25) |
| Greater than 85 | 0.87 | 0.86 | 0.01 (-0.05-0.07) | 0.88 | 0.85 | 0.03 (-0.07-0.14) | 0.86 | 0.86 | 0.00 (-0.08-0.07) |

*AR: absolute risk of death; ARD: absolute risk difference between CCI groups*

**sTable 14:** Univariate and adjusted associations between pre-stroke multimorbidity and all-cause mortality at 10 years follow-up, stratified by aetiological subtype of ischaemic stroke

| **Model # / Adjustments** | | ***Set A: Hazard Ratios (95%CI) for CCI ≥ 2 vs 0 (unweighted/counts)*** | | | | | | | |
| --- | --- | --- | --- | --- | --- | --- | --- | --- | --- |
|  |  | **Cardioembolic**  **(n=644)** | | **Large Artery Disease**  **(n=215)** | **Small Vessel Disease**  **(n=276)** | **Undetermined**  **(n=522)** | **Unknown**  **(n=332)** | | **Multiple or Other (N=149)** |
| Model 1 | *Crude* | 1.97 (1.59-2.45)*** | | 2.17 (1.33-3.53)*** | 4.13 (2.59-6.57)*** | 2.57 (1.84-3.57)*** | 1.45 (1.09-1.92)* | | 3.35 (1.94-5.79)*** |
| Model 2 | *Age, Sex* | 1.82 (1.47-2.27)*** | | 1.75 (1.07-2.87)* | 3.11 (1.95-4.97)*** | 1.70 (1.21-2.37)** | 1.39 (1.05-1.85)* | | 2.29 (1.31-3.99)** |
| Model 3A | *+ IMD* | 1.84 (1.48-2.28)*** | | 1.70 (1.04-2.80)* | 3.08 (1.93-4.93)*** | 1.58 (1.12-2.22)** | 1.38 (1.04-1.84)* | | 2.30 (1.32-4.02)** |
| Model 3B | *+ Smoking* | 1.83 (1.47-2.28)*** | | 1.75 (1.06-2.90)* | 3.15 (1.97-5.03)*** | 1.67 (1.19-2.34)** | 1.40 (1.05-1.96)* | | 2.27 (1.30-3.96)** |
| Model 3C | *+ Depression/Anxiety* | 1.82 (1.47-2.27)*** | | 1.80 (1.09-2.96)* | 3.19 (1.99-5.11)*** | 1.73 (1.24-2.43)** | 1.40 (1.05-1.87)* | | 2.29 (1.32-3.98)** |
| Model 3C | *+ Hypertension* | 1.82 (1.46-2.26)*** | | 1.78 (1.08-2.94)* | 3.28 (2.04-5.29)*** | 1.72 (1.22-2.43)** | 1.39 (1.04-1.85)* | | 2.32 (1.33-4.04)** |
| Model 3D | *+ Hyperlipidaemia* | 1.83 (1.47-2.28)*** | | 1.84 (1.11-3.04)* | 3.28 (1.99-5.42)*** | 1.69 (1.20-2.38)** | 1.38 (1.03-1.85)* | | 2.31 (1.30-4.12)** |
| Model 3E | *+ Atrial Fibrillation* | 1.82 (1.46-2.26)*** | | 1.71 (1.04-2.82)* | 3.11 (1.95-4.97)*** | 1.70 (1.21-2.37)** | 1.32 (0.98-1.76) | | 2.25 (1.29-3.92)** |
| Model 3F | *+ NIHSS* | 1.92 (1.54-2.38)*** | | 2.26 (1.34-3.80)** | 3.14 (1.97-5.03)*** | 1.84 (1.31-2.58)*** | 1.32 (0.99-1.76) | | 2.24 (1.28-3.92)** |
| Model 3G | *+ mRS* | 1.63 (1.31-2.04)*** | | 1.41 (0.85-2.36) | 3.25 (2.03-5.19)*** | 1.56 (1.12-2.19)** | 1.31 (0.98-1.75) | | 1.69 (0.94-3.02) |
| Model 4 | *+ All* | 1.72 (1.37-2.16)*** | | 1.66 (0.94-2.93) | 3.56 (2.14-5.91)*** | 1.68 (1.17-2.40)** | 1.22 (0.89-1.65) | | 1.67 (0.88-3.16) |
|  | | |  | | | | | | |
| **Model # / Adjustments** | | | ***Set B: Hazard Ratios (95%CI) for CCI ≥ 2 vs 0 (weighted/points)*** | | | | | | |
|  |  |  | **Cardioembolic**  **(n=644)** | **Large Artery Disease**  **(n=215)** | **Small Vessel Disease**  **(n=276)** | **Undetermined**  **(n=522)** | | **Unknown**  **(n=332)** | **Multiple or Other (N=149)** |
| Model 1 | *Crude* | | 1.91 (1.55-2.34)*** | 2.21 (1.39-3.50)*** | 3.12 (2.02-4.84)*** | 2.13 (1.55-2.94)*** | | 1.46 (1.11-1.91)** | 3.12 (1.82-5.31)*** |
| Model 2 | *Age, Sex* | | 1.70 (1.38-2.10)*** | 1.70 (1.07-2.71)* | 2.49 (1.60-3.86)*** | 1.44 (1.04-1.99)* | | 1.43 (1.09-1.88)* | 2.16 (1.26-3.68)** |
| Model 3A | *+ IMD* | | 1.72 (1.40-2.13)*** | 1.71 (1.07-2.73)* | 2.44 (1.57-3.78)*** | 1.35 (0.97-1.88) | | 1.41 (1.07-1.86)* | 2.16 (1.26-3.70)** |
| Model 3B | *+ Smoking* | | 1.71 (1.38-2.10)*** | 1.70 (1.06-2.72)* | 2.57 (1.66-3.99)*** | 1.42 (1.03-1.97)* | | 1.43 (1.09-1.88)* | 2.14 (1.25-3.66)** |
| Model 3C | *+ Depression/Anxiety* | | 1.70 (1.38-2.10)*** | 1.75 (1.10-2.81)* | 2.52 (1.62-3.91)*** | 1.44 (1.04-1.99)* | | 1.43 (1.09-1.88)* | 2.13 (1.25-3.63)** |
| Model 3C | *+ Hypertension* | | 1.70 (1.37-2.09)*** | 1.72 (1.08-2.76)* | 2.58 (1.66-4.02)*** | 1.45 (1.04-2.00)* | | 1.43 (1.08-1.88)* | 2.17 (1.27-3.71)** |
| Model 3D | *+ Hyperlipidaemia* | | 1.71 (1.38-2.10)*** | 1.75 (1.10-2.81)* | 2.50 (1.59-3.93)*** | 1.43 (1.03-1.98)* | | 1.42 (1.07-1.87)* | 2.14 (1.23-3.70)** |
| Model 3E | *+ Atrial Fibrillation* | | 1.69 (1.37-2.09)*** | 1.70 (1.04-2.69)* | 2.48 (1.60-3.85)*** | 1.44 (1.04-1.99)* | | 1.36 (1.03-1.80)* | 2.13 (1.24-3.64)** |
| Model 3F | *+ NIHSS* | | 1.76 (1.42-2.17)*** | 2.14 (1.31-3.48)* | 2.50 (1.62-3.88)*** | 1.55 (1.12-2.15)** | | 1.33 (1.01-1.76)* | 2.11 (1.23-3.63)** |
| Model 3G | *+ mRS* | | 1.58 (1.28-1.95)*** | 1.40 (0.86-2.27) | 2.60 (1.67-4.03)*** | 1.40 (1.01-1.93)* | | 1.36 (1.03-1.79)* | 1.67 (0.96-2.92) |
| Model 4 | *+ All* | | 1.65 (1.33-2.04)*** | 1.68 (0.98-2.87) | 2.67 (1.70-4.21)*** | 1.46 (1.04-2.04)* | | 1.25 (0.94-1.67) | 1.63 (0.89-2.98) |

*Expressed as hazard ratio (95%CI) for CCI ≥ 2 vs 0 (weighted)*

**p<0.05 **p<0.01 ***p<0.001; CCI: Charlson Comorbidity Index, IMD: Index of Multiple Deprivation, NIHSS: National Institutes of Health Stroke Scale, mRS: Modified Rankin Scale.*

**sTable 15:** Univariate and adjusted associations between pre-stroke multimorbidity and all-cause mortality at 10 years follow-up in the Low Risk THRIVE Score group (original and adjusted), stratified by aetiological subtype of ischaemic stroke

| **Model # / Adjustments** | | **Aetiological Subtypes (TOAST)** | | | | | |
| --- | --- | --- | --- | --- | --- | --- | --- |
|  |  | ***Cardioembolic*** | ***Large Artery Disease*** | ***Small Vessel Disease*** | ***Undetermined*** | ***Unknown*** | ***Multiple or Other*** |
|  |  | ***Hazard Ratios (95%CI) for CCI ≥ 2 vs 0 (unweighted/counts)*** | | | | | |
| **THRIVE Adjusted Scoring** | |  |  |  |  |  |  |
| Model 1 | *Crude* | 2.15 (1.05-4.39)* | 2.89 (1.22-6.84)* | 4.96 (2.58-9.54)*** | 3.61 (2.20-5.91)*** | 1.42 (0.69-2.90) | 3.52 (1.12-11.18)* |
| Model 2 | *Adj. Age, Sex* | 1.86 (0.90-3.84) | 1.98 (0.79-4.96) | 4.20 (2.17-8.11)*** | 2.56 (1.52-4.30)*** | 1.45 (0.71-2.98) | 1.87 (0.55-6.34) |
| **THRIVE Original Scoring** | |  |  |  |  |  |  |
| Model 1 | *Crude* | 2.18 (1.25-3.79)** | 2.17 (1.03-4.61)* | 4.73 (2.57-8.56)*** | 3.45 (2.18-5.48)*** | 1.73 (1.03-2.89)* | 3.19 (1.12-9.10)* |
| Model 2 | *Adj. Age, Sex* | 1.82 (1.04-3.20)* | 1.79 (0.82-3.90) | 4.75 (2.53-8.94)*** | 2.34 (1.45-3.80)** | 1.66 (0.99-2.78) | 1.43 (0.47-4.32) |
|  |  | ***Hazard Ratios (95%CI) for CCI ≥ 2 vs 0 (weighted/points)*** | | | | | |
| **THRIVE Adjusted Scoring** | |  |  |  |  |  |  |
| Model 1 | *Crude* | 1.82 (0.95-3.46) | 2.17 (0.94-5.01) | 2.81 (1.55-5.07)** | 2.43 (1.51-3.92)*** | 1.30 (0.70-2.43) | 3.47 (1.16-10.30)* |
| Model 2 | *Adj. Age, Sex* | 1.52 (0.79-2.91) | 1.40 (0.59-3.36) | 2.20 (1.21-3.98)** | 1.66 (1.02-2.71)* | 1.41 (0.75-2.63) | 2.11 (0.68-6.59) |
| **THRIVE Original Scoring** | |  |  |  |  |  |  |
| Model 1 | *Crude* | 1.93 (1.16-3.23)* | 2.05 (1.02-4.11)* | 2.79 (1.58-4.94)*** | 2.29 (1.47-3.59)*** | 1.54 (0.97-2.45) | 2.96 (1.12-7.80)* |
| Model 2 | *Adj. Age, Sex* | 1.55 (0.92-2.60) | 1.59 (0.78-3.23) | 2.49 (1.41-4.41)** | 1.56 (0.99-2.48) | 1.56 (0.98-2.48) | 1.78 (0.65-4.86) |

*Original THRIVE scoring includes the following NIHSS categories (NIHSS ≤ 10: 0 points, 11-20: 2 points, ≥ 21: 4 points) based on NIHSS on presentation. We also used adjusted THRIVE categories with altered NIHSS scoring (NIHSS ≤ 4: 0 points, 5-9: 2 points, ≥ 10: 4 points), as the present study included sub-acute phase NIHSS as opposed to NIHSS on presentation. CCI: Charlson Comorbidity Index; TOAST: Trial of Org 10172 in Acute Stroke Treatment*

**sFigure 1:** Kaplan-Meier curves for vascular and non-vascular mortality up to 10 years post-event

**Vascular Death**

**Non-Vascular Death**

*X^2^*=107.0

*p*<0.001

*X^2^*=58.5

*p*<0.001

**Cumulative incidence**

**
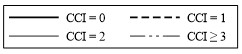
**

**Years since index event**

**Years since index event**

*Unweighted CCI was used for the present figure. Results of the Log-Rank test shown in respective panels. CCI: Charlson Comorbidity Index.*

**sFigure 2:** Kaplan-Meier curves for all-cause mortality in the overall cohort and across original THRIVE groups using the unweighted Charlson Comorbidity Index

*
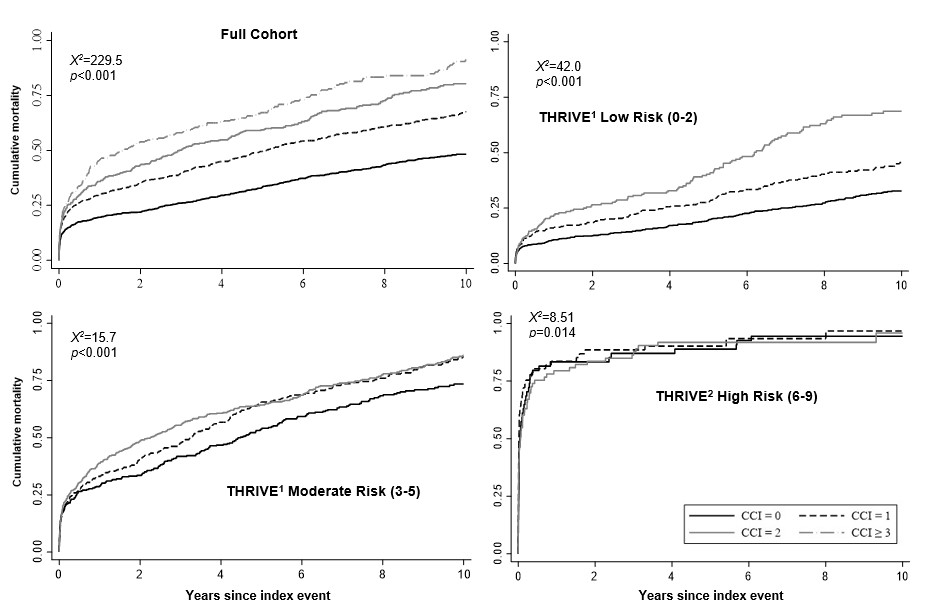
*

*X^2^*=1.25

*p*=0.53

**THRIVE^1^ High Risk (≥6)**

*Results of corresponding log-rank tests shown in respective Kaplan-Meier plots.^1^Original THRIVE scoring includes the following NIHSS categories (NIHSS ≤ 10: 0 points, 11-20: 2 points, ≥ 21: 4 points) based on NIHSS on presentation, and was used for the present figure. A figure including adjusted THRIVE categories with altered NIHSS scoring (NIHSS ≤ 4: 0 points, 5-9: 2 points, ≥ 10: 4 points), as the present study included sub-acute phase NIHSS as opposed to NIHSS on presentation, is found in the main text (Figure 1). Only 3 CCI (unweighted) categories were used for the present figure for the panels using different THRIVE Scores, with grey representing all patients with 2 or more CCI comorbidities.* *The present figure used the unweighted CCI.*

**sFigure 3:** Kaplan-Meier curves for all-cause mortality in the THRIVE low risk group at 90 days, 1-year, and 10-years follow-up using the unweighted Charlson Comorbidity Index

**THRIVE^1^ Low Risk (0-2)**

**10-year Follow-Up**

**THRIVE^1^ Low Risk (0-2)**

**1-year Follow-Up**

**THRIVE^1^ Low Risk (0-2)**

**90-days Follow-Up**

*X^2^*=4.34

*p*=0.11

*X^2^*=16.9

*p*<0.001

*X^2^*=79.7

*p*<0.001

**Cumulative mortality**

*
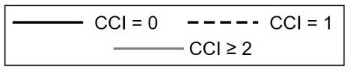
*

*X^2^*=67.6

*p*<0.001

*X^2^*=13.1

*p*<0.01

*X^2^*=3.13

*p*=0.21

**Days since index event**

**Years since index event**

**Years since index event**

**Cumulative mortality**

**THRIVE^2^ Low Risk (0-2)**

**10-year Follow-Up**

**THRIVE^2^ Low Risk (0-2)**

**1-year Follow-Up**

**THRIVE^2^ Low Risk (0-2)**

**90-days Follow-Up**

*Unweighted CCI was used for the present figure. Results of the Log-Rank test shown in respective panels. CCI: Charlson Comorbidity Index.*

**sFigure 4:** Kaplan-Meier curves for all-cause mortality up to 10 years post-event, stratified by deprivation level

**

**

**Below Median of IMD Scores (Less Deprived)**

**Above Median of IMD Scores (More Deprived)**

*X^2^*=115.4

*p*<0.001

*X^2^*=102.2

*p*<0.001

**Cumulative mortality**


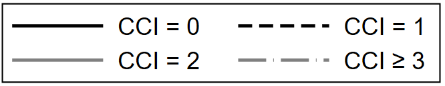


**Years since index event**

**Years since index event**

*Unweighted CCI was used for the present figure. Results of the Log-Rank test shown in respective panels. CCI: Charlson Comorbidity Index; IMD: Index of Multiple Deprivation*

**sFigure 5:** Kaplan-Meier curves for all-cause mortality up to 10 years post-event, stratified by smoking history

**

**Never Smoked**

**Ever Smoked**

*X^2^*=123.3

*p*<0.001

*X^2^*=103.5

*p*<0.001

**Cumulative mortality**


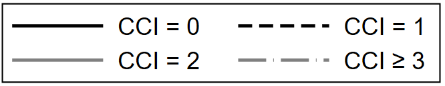


**Years since index event**

**Years since index event**

*Unweighted CCI was used for the present figure. Results of the Log-Rank test shown in respective panels. CCI: Charlson Comorbidity Index.*

**sFigure 6:** Kaplan-Meier curves for all-cause mortality up to 10 years post-event, stratified by history of depression or anxiety

**

**History of Depression or Anxiety**

**No History of Depression or Anxiety**

*X^2^*=63.6

*p*<0.001

*X^2^*=156.3

*p*<0.001

**Cumulative mortality**


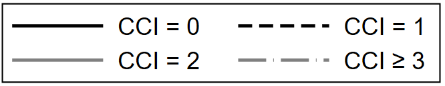


**Years since index event**

**Years since index event**

*Unweighted CCI was used for the present figure. Results of the Log-Rank test shown in respective panels. CCI: Charlson Comorbidity Index.*

**sFigure 7:** Kaplan-Meier curves for all-cause mortality up to 10 years post-event, stratified by history of hypertension

**

**No Prior Hypertension**

**Prior Hypertension**

*X^2^*=89.4

*p*<0.001

*X^2^*=120.4

*p*<0.001


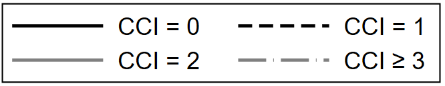


*Unweighted CCI was used for the present figure. Results of the Log-Rank test shown in respective panels. CCI: Charlson Comorbidity Index.*

**sFigure 8:** Kaplan-Meier curves for all-cause mortality up to 10 years post-event, stratified by history of hyperlipidaemia

**No Prior Hyperlipidaemia**

**Prior Hyperlipidaemia**

*X^2^*=59.4

*p*<0.001

*X^2^*=175.6

*p*<0.001


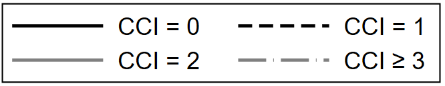


*Unweighted CCI was used for the present figure. Results of the Log-Rank test shown in respective panels. CCI: Charlson Comorbidity Index.*

**sFigure 9:** Kaplan-Meier curves for all-cause mortality up to 10 years post-event, stratified by history of atrial fibrillation

**Prior Atrial Fibrillation**

**No Prior Atrial Fibrillation**

*X^2^*=31.7

*p*<0.001

*X^2^*=133.6

*p*<0.001


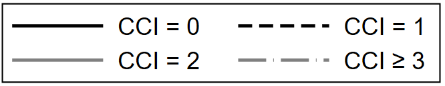


*Unweighted CCI was used for the present figure. Results of the Log-Rank test shown in respective panels. CCI: Charlson Comorbidity Index.*

**sFigure 10:** Kaplan-Meier curves for all-cause mortality up to 10 years post-event, stratified by aetiological subtypes

**

**Large Artery Disease**

**Small Vessel Disease**

*X^2^*=40.7

*p*<0.001

*X^2^*=11.4

*p*<0.001

**Cumulative mortality**

**Multiple or Other**

**Undetermined**

*X^2^*=30.0

*p*<0.001

*X^2^*=33.2

*p*<0.001

**Cumulative mortality**

*
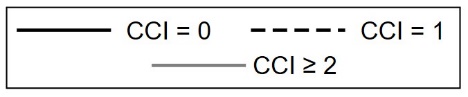
*

**Years since index event**

**Years since index event**

**Unknown Aetiology**

**Cardioembolic Stroke**

*X^2^*=42.0

*p*<0.001

*X^2^*=8.4

*p*=0.015

**Cumulative mortality**

*
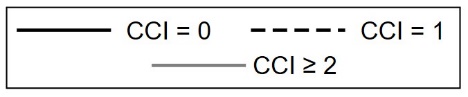
*

**Years since index event**

**Years since index event**

*Unweighted CCI was used for the present figure. Results of the Log-Rank test shown in respective panels. CCI: Charlson Comorbidity Index.*

**sFigure 11:** Distribution of 2298 stroke or transient ischaemic attack patients in the Oxford Vascular Study (2002-2012) by the number of Charlson and Elixhauser Index comorbidities.

Unweighted Scores: *R*^2^=0.53

Weighted Scores: *R*^2^=0.39

*As data on all Elixhauser Index (EI) comorbidities were not available in the present cohort for the current analysis, we used another OXVASC Cohort (patients with transient ischaemic attack or stroke ascertained from 2002-2012) to compare the number of CCI and EI comorbidities. We also correlated both the weighted and unweighted versions of the CCI and EI using Pearson Correlations (both p<0.001; R^2^ values presented above).*

*CCI: Charlson Comorbidity Index, EI: Elixhauser Index*
